# Adiposome Proteomics Uncover Molecular Signatures of Cardiometabolic Risk in Obese Individuals

**DOI:** 10.3390/proteomes13030039

**Published:** 2025-08-26

**Authors:** Mohamed Saad Rakab, Monica C. Asada, Imaduddin Mirza, Mohammed H. Morsy, Amro Mostafa, Francesco M. Bianco, Mohamed M. Ali, Chandra Hassan, Mario A. Masrur, Brian T. Layden, Abeer M. Mahmoud

**Affiliations:** 1 Faculty of Medicine, Mansoura University, Mansoura 35516, Egypt; mohamedrikab2000@gmail.com; 2 Department of Medicine, Division of Endocrinology, Diabetes, and Metabolism, College of Medicine, University of Illinois Chicago, Chicago, IL 60612, USA; mchen234@uic.edu (M.C.A.); mmirza24@uic.edu (I.M.); mmorsy3@uic.edu (M.H.M.); blayde1@uic.edu (B.T.L.); 3 Department of Pharmacology, College of Medicine, University of Illinois Chicago, Chicago, IL 60612, USA; amost2@uic.edu; 4 Department of Surgery, College of Medicine, University of Illinois Chicago, Chicago, IL 60612, USA; biancofm@uic.edu (F.M.B.); chandrar@uic.edu (C.H.); mmasrur@uic.edu (M.A.M.); 5 Department of Biobehavioral Nursing Science, College of Nursing, University of Illinois Chicago, Chicago, IL 60612, USA; mali37@uic.edu; 6 Department of Medicine, Jesse Brown Veterans Affairs Medical Center, Chicago, IL 60612, USA; 7 Department of Kinesiology and Nutrition, College of Applied Health Sciences, University of Illinois Chicago, Chicago, IL 60612, USA

**Keywords:** adiposomes, extracellular vesicles, obesity, proteomics, visceral adipose tissue, cardiometabolic disease, inflammation, endothelial dysfunction, lipid metabolism, machine learning

## Abstract

Background: Adipose-derived extracellular vesicles (adiposomes) are emerging as key mediators of inter-organ communication, yet their molecular composition and role in obesity-related pathophysiology remain underexplored. This study integrates clinical phenotyping with proteomic analysis of visceral adipose-derived adiposomes to identify obesity-linked molecular disruptions. Methods: Seventy-five obese and forty-seven lean adults were extensively profiled for metabolic, inflammatory, hepatic, and vascular parameters. Adiposomes isolated from visceral fat underwent mass spectrometry-based proteomic analysis, followed by differential abundance, pathway enrichment, regulatory network modeling, and clinical association testing. Results: Obese individuals exhibited widespread cardiometabolic dysfunction. Proteomics revealed 64 adiposomal proteins with differential abundance. Upregulated proteins (e.g., CRP, C9, APOC1) correlated with visceral adiposity, systemic inflammation, and endothelial dysfunction. In contrast, downregulated proteins (e.g., ADIPOQ, APOD, TTR, FGB, FGG) were associated with enhanced nitric oxide bioavailability and vascular protection, suggesting loss of homeostatic signaling. Network analyses identified TNF and IL1 as key upstream regulators driving inflammatory and oxidative stress pathways. Decision tree and random forest models accurately classified obesity, hypertension, diabetes, dyslipidemia, and hepatic steatosis (AUC = 0.908–0.994), identifying predictive protein signatures related to complement activation, inflammation, and lipid transport. Conclusion: Obesity alters adiposome proteomic cargo, reflecting and potentially mediating systemic inflammation, metabolic dysregulation, and vascular impairment.

## 1. Introduction

Obesity is a significant global health issue affecting over 650 million adults worldwide and is a primary contributor to the rising prevalence of type 2 diabetes, cardiovascular disease, nonalcoholic fatty liver disease, musculoskeletal diseases, and certain malignancies [1]. The defining characteristic of obesity is the accumulation of excess fat; nevertheless, the effect of this fat extends beyond mere increases in body mass, leading to a persistent, low-grade inflammatory state and extensive metabolic dysregulation [2]. There has been a growing focus on the biological impact of this expanding adipose tissue mass on systemic homeostasis and disease risk.

Adipose tissue is now recognized as an active secretory organ that produces a myriad of bioactive factors, collectively known as adipokines, such as leptin, adiponectin, resistin, and inflammatory cytokines such as TNF-α and IL-6 [3]. These molecules act in paracrine and endocrine manners to regulate energy metabolism, inflammation, and other biological processes. Expanding this secretory repertoire, adipose-derived extracellular vesicles (EVs), referred to as adiposomes, have recently emerged as an additional and highly dynamic component of the adipose secretome [4]. In parallel, EVs have rapidly gained recognition as critical mediators of metabolic regulation and inter-organ communication. Initially regarded as cellular debris, EVs are now known to be complex carriers of bioactive molecules such as proteins, lipids, and nucleic acids [5]. Early research predominantly focused on the microRNA cargo of EVs, revealing their ability to modulate inflammatory responses and insulin signaling pathways [6,7]. More recently, interest has shifted toward their protein composition and the role of this protein cargo in cellular signaling, immune regulation, and metabolic homeostasis [8].

The adipose tissue microenvironment is highly responsive to metabolic stress and undergoes profound remodeling in the setting of obesity, as demonstrated in both animal models and human studies. This remodeling is driven by sustained transcriptional and epigenetic reprogramming in adipocytes, progenitor cells, endothelial cells, and macrophages, resulting in impaired metabolic regulation, enhanced fibrotic deposition, and chronic low-grade inflammation [9,10]. In this context of adipose tissue dysfunction, adiposomes have gained attention as key mediators of inter-organ communication. By transporting bioactive cargo, adiposomes are expected to influence peripheral insulin sensitivity, inflammatory signaling, and vascular homeostasis, thereby playing a potentially central role in the progression of obesity-related metabolic diseases [11]. Our experimental studies have shown that adiposomes derived from obese individuals contribute to endothelial dysfunction by compromising caveolar structure and disrupting nitric oxide signaling within endothelial cells [12].

We also reported distinct lipidomic signatures in adiposomes from obese versus lean individuals, marked by elevated levels of ceramides and free fatty acids, which may contribute to their adverse metabolic and vascular effects [13]. While these lipid alterations underscore the pathogenic potential of adiposomes, lipids represent only one facet of their bioactive cargo. Given that extracellular vesicle function is often dictated by the coordinated action of both lipid and protein components [14], we have turned our focus in this study to adiposome-associated proteins. By characterizing the proteomic landscape of adiposomes, we aim to uncover additional mechanistic mediators and regulatory networks through which adiposomes may influence obesity-related pathophysiology.

Proteomic studies have highlighted depot-specific differences in adipocyte metabolism, revealing alterations in lipid handling, extracellular vesicle biogenesis, inflammation, and fibrosis that reflect the complexity of adipose tissue remodeling in obesity [15]. However, the proteomic landscape of adiposomes remains largely uncharted. Most existing work has focused on selected candidate molecules or relied on in vitro models, leaving a gap in comprehensive analyses of human adiposomes in the context of obesity [16]. Given the pivotal role of visceral adipose tissue in metabolic regulation and disease progression [17], profiling the molecular cargo of adiposomes derived from this depot may offer critical insight into the signaling pathways that drive obesity-related dysfunction.

In this study, we conducted high-resolution proteomic profiling of adiposomes isolated from visceral adipose tissue in a well-characterized cohort of obese and lean individuals. Using mass spectrometry coupled with differential abundance analysis, network-based pathway modeling, and upstream regulatory inference, we sought to (1) define the proteomic alterations in adiposomes associated with obesity, (2) identify functional networks and upstream signals linked to these changes, and (3) determine how specific adiposome proteins correlate with key clinical and metabolic outcomes. Our findings reveal a distinct obesity-associated adiposome proteome enriched in inflammatory, immune, and structural remodeling signatures, supporting a role for adiposomes as both biomarkers and active contributors to obesity-related cardiometabolic risk.

## 2. Materials and Methods

### 2.1. Subject Enrollment

This study was conducted at the University of Illinois Hospital in Chicago and included 75 obese adults (BMI ≥ 30 kg/m^2^; 45 women and 30 men) scheduled for sleeve gastrectomy, along with 47 lean adults (BMI < 25 kg/m^2^; 26 women and 21 men) undergoing elective surgeries such as hernia repair. The obese cohort comprised 42 African American, 20 Hispanic, and 13 Non-Hispanic White individuals, while the lean group included 23 African American, 15 Hispanic, and 9 Non-Hispanic White participants. All subjects were between 22 and 50 years of age. Blood samples and physiological assessments were obtained 2–3 weeks prior to surgery, and visceral adipose tissue (VAT) samples were collected intraoperatively. Exclusion criteria included pregnancy, active smoking, prior bariatric surgery, and current diagnoses of liver, kidney, or heart failure, cancer, or autoimmune disease. The study adhered to the principles of the Declaration of Helsinki and received approval from the Institutional Review Board of the University of Illinois at Chicago (protocol #2021-1113, approved 25 October 2021). Written informed consent was obtained from all participants before enrollment.

### 2.2. Cardiometabolic Risk Measurements

Anthropometric measurements, including body weight and body mass index (BMI), were obtained for all participants. Body composition, specifically fat mass and lean mass percentages, was assessed using dual-energy X-ray absorptiometry (DEXA) with the iDXA system (GE Healthcare). Fasting blood samples were collected and analyzed for glucose, insulin, and hemoglobin A1c levels using previously validated protocols [18]. Insulin resistance was estimated using the homeostatic model assessment of insulin resistance (HOMA-IR), calculated as: fasting insulin (μU/mL) × fasting glucose (mmol/L) ÷ 22.5 [19]. Serum lipid profiles, including total cholesterol, low-density lipoprotein (LDL), high-density lipoprotein (HDL), and triglycerides, were measured using standardized enzymatic assays (Roche Diagnostics, Indianapolis, IN, USA) according to established protocols [18]. Liver function was evaluated via routine clinical laboratory testing of alanine aminotransferase (ALT), aspartate aminotransferase (AST), bilirubin, albumin, and alkaline phosphatase levels.

Plasma nitric oxide (NO) levels were quantified using a nitrate/nitrite colorimetric assay kit (Cayman Chemical, Ann Arbor, MI, USA) following our previously published protocol [20]. Circulating inflammatory markers, including interleukin-6 (IL-6) and high-sensitivity C-reactive protein (CRP), were measured using immunoassays (R&D Systems, Minneapolis, MN, USA). Serum adiponectin concentrations were assessed using a commercial ELISA kit (Thermo Fisher Scientific, Waltham, MA, USA; catalog #KHP004). Hepatic fat accumulation was assessed noninvasively using the Attenuation Imaging (ATI) module on the Aplio i900 ultrasound system (Canon Medical Systems, Melville, NY, USA). A low-frequency curvilinear transducer was used to visualize the right hepatic lobe and the ATI software V7.0 automatically calculated the attenuation coefficient (dB/cm/MHz) across standardized regions of interest, providing a quantitative index of hepatic steatosis.

### 2.3. Adiposome Isolation and Protein Extraction

Adipose tissue biopsies were processed using previously established protocols [12]. Briefly, freshly excised visceral adipose tissue was rinsed with sterile Medium 199 (Gibco, Waltham, MA, USA), minced into small fragments, and subjected to enzymatic digestion in a solution containing Type I collagenase (Worthington) and 4% bovine serum albumin (BSA) (catalog no. A2153, Millipore Sigma, Rockville, MD, USA) in Medium 199. The resulting cell suspension was filtered and centrifuged at 500× *g* to isolate mature adipocytes from the floating layer. Isolated adipocytes were transferred onto membrane inserts (Corning^®^ 24 mm Transwell^®^ with 0.4 µm Pore Polyester Membrane Insert, Sterile, catalog no. 3450, Life Sciences, Huntsville, AL, USA) and cultured in Medium 199 supplemented with 5% exosome-depleted fetal bovine serum (FBS, catalog no. A2720801; ThermoFisher Scientific, Waltham, MA, USA) and 1% penicillin-streptomycin. After 24–48 h of incubation, the conditioned medium was collected and processed by sequential centrifugation, initially at 1000× *g* for 5 min and then at 15,000× *g* for 15 min. The supernatant was filtered through a 0.45 µm membrane and ultracentrifuged at 150,000× *g* for 2 h to pellet adiposomes. The adiposome pellet was resuspended and characterized using a Nanoparticle Tracking Analyzer (NanoSight NS300; Malvern Instruments, Malvern, UK).

Protein extraction from isolated adiposomes was performed using RIPA buffer, and total protein content was quantified with the Pierce BCA Protein Assay Kit (Thermo Fisher Scientific). Proteins (25 μg) were resolved by SDS-PAGE on 4–12% Bis-Tris gradient gels (catalog no. 3450124) and transferred to polyvinylidene fluoride (PVDF) membranes (catalog no. 1620177) (Bio Rad Laboratories; Hercules, CA, USA). Membranes were probed overnight at 4 °C with primary antibodies against CD9 (E8L5J Rabbit mAb catalog no. 98327, Cell Signaling Biotechnology, Danvers, MA, USA), CD81 (M38 mouse mAb catalog no. 10630D, ThermoFisher Scientific), CD63 (Goat polyclonal Ab catalog no. LS-C204227, Lifespan Biosciences, Inc., Seattle, WA, USA), PPARγ (D8I3Y Mouse mAb catalog. No. 95128, Cell Signaling Biotechnology, Danvers, MA, USA), adiponectin (C45B10 Rabbit mAb catalog no. 2789, Cell Signaling Biotechnology, Danvers, MA, USA), apolipoprotein B (APOB; Rabbit mAb EPR27473-49, abcam, Waltham, MA, USA), and fatty acid-binding protein 4 (FABP4; Goat polyclonal Ab catalog no. ab195657). All primary antibodies were used at a 1:500 dilution. Target proteins had distinct molecular weights, enabling simultaneous incubation with multiple antibodies on a single blot. A molecular weight protein ladder was run alongside the samples to assist in accurate band identification. Following primary antibody incubation, membranes were incubated for 2 h at room temperature with species-specific secondary antibodies conjugated to distinct fluorophores (1) IRDye^®^ 800CW Goat anti-Rabbit IgG1 (P/N: 926-32211), (2) IRDye 680LT Goat anti-Mouse (P/N: 926-68020), and (3) VRDye™ 549 Donkey anti-Goat (P/N: 926-54014). All secondary antibodies were from LI-COR Biosciences (Lincoln, NE, USA) and were used at a 1:10,000 dilution. This approach enabled concurrent detection of multiple targets on a single membrane using the Odyssey multi-channel imaging system (LI-COR Biosciences).

### 2.4. Adiposome Protein Analysis

Protein samples were centrifuged to remove insoluble precipitates and the resulting clear supernatant was collected and quantified using the Qubit Protein Assay (Catalog number Q33211) following the manufacturer’s protocol (ThermoFisher Scientific, Waltham, MA, USA). To minimize interference from highly abundant plasma proteins, such as albumin and complement components, 300 µg of total protein was incubated with 200 µL of Top14 Depletion resin (ThermoFisher Scientific, Catalog no. A36372) for 15 min. After depletion, the samples were centrifuged, yielding approximately 110 µL of supernatant, which was re-quantified before downstream processing. To account for inter-individual variability in adipocyte content, equivalent amounts of adipose tissue (500 mg per subject) were processed for adiposome isolation. The isolated adiposome yield was normalized per mg of tissue and confirmed by total protein concentration using the Qubit assay. All downstream proteomic analyses used equal total protein input (30 µg per sample after depletion), ensuring comparable representation of adiposome protein cargo between groups as previously published [21,22,23,24,25]. For proteomic analysis, 30 µg of depleted protein from each sample was processed using the Filter-Aided Sample Preparation (FASP) protocol as previously described by Wiśniewski et al. [26] with 10 kDa molecular weight cutoff filters (VWR^®^ Centrifugal Filters, Catalog no. 82031-348, Avantor, Inc., Radnor, PA, USA). Samples were reduced with 20 mM DTT (dithiothreitol) for 30 min, alkylated in the dark with 50 mM IAA (iodoacetamide) for 40 min, and washed with 8 M urea in 0.1 M ammonium bicarbonate (ABC). After equilibration, overnight trypsin digestion was performed at 37 °C using a 1:30 enzyme-to-protein ratio. Peptides were eluted by centrifugation, washed with ABC and NaCl, desalted using Oasis PRiME HLB cartridges (Waters, Catalog no. 186008055, Molford, MA, USA), and dried under vacuum. The final peptide products were reconstituted in 75 µL of 5% acetonitrile with 0.1% formic acid and quantified before mass spectrometry using the Pierce Quantitative Colorimetric Peptide Assay (Thermo Fisher, Catalog no. 23275).

A total of 122 peptide samples were processed across 17 TMT10-plex batches, with each sample labeled using TMT10-plex (Tandem Mass Tag labeling Isobaric Label Reagent Set, Thermo Fisher Scientific, catalog no. 90111), according to the manufacturer’s protocol. Labeling reactions were quenched with 4 µL hydroxylamine, after which the samples were pooled and desalted using Oasis HLB µElution Plates (Waters Catalog no. 186001828BA). From each pooled batch, 200 µg of peptides were subjected to high-pH reversed-phase (HPRP) fractionation on an XBridge BEH C18 column. To reduce sample complexity, every 11th fraction was concatenated, resulting in 10 combined fractions. These were subsequently dried and reconstituted in 50 µL of 5% acetonitrile with 0.1% formic acid for LC-MS analysis.

Approximately 1 µg of peptide from each pooled fraction was analyzed using a Q Exactive HF mass spectrometer (Thermo Fisher Scientific) coupled to an UltiMate 3000 RSLCnano system. Peptides were first trapped and then separated on Waters nanoEase and BEH C18 columns at a flow rate of 300 nL/min over a 105-min linear gradient using solvent A (0.1% formic acid in water) and solvent B (0.1% formic acid in 80% acetonitrile). MS1 scans were acquired at 120,000 resolution across a 350–1400 *m*/*z* range. The top 15 most intense precursor ions (charge states 2–5) were selected for data-dependent MS/MS fragmentation using higher-energy collisional dissociation (HCD) at 32% normalized collision energy (NCE), with dynamic exclusion enabled for 30 s. MS2 scans were acquired at 60,000 resolution, with AGC targets set to 3E6 for MS1 and 1E5 for MS2, and maximum injection times of 50 ms and 120 ms, respectively.

Spectral data were processed using Mascot Daemon (v2.6.0) against the UniProt Human database (version 08/11/2020), with a parent ion mass tolerance of 10 ppm (Supplementary Table S3). Carbamidomethylation of cysteine was set as a fixed modification, while oxidation (M) and deamidation (N/Q) were included as variable modifications. Peptide and protein quantification were performed using Scaffold DDA (v6.0.1), which also applied TMT reporter ion purity corrections. Protein identifications were filtered using a 5% false discovery rate (FDR) threshold and required at least one confidently identified peptide per protein.

While our non-targeted proteomic analysis was performed using a bottom-up mass spectrometry approach, it is important to note that this method does not enable full proteoform resolution. Since proteins are digested into peptides prior to analysis, information about the co-occurrence of post-translational modifications (PTMs), alternative splicing events, or sequence variants on the same protein molecule is lost. Therefore, our data represent protein-level and peptide-level identifications, but not intact proteoforms.

### 2.5. Vascular Function Assessment

Brachial artery flow-mediated dilation (FMD) was evaluated using a high-resolution vascular ultrasound system (Aplio i900; Canon Ultrasound Systems, Melville, NY, USA). A blood pressure cuff was positioned on the participant’s forearm and inflated to 220 mmHg for 5 min to induce transient ischemia. Arterial diameter was measured 1 min before cuff inflation (baseline) and again 5 min after cuff release during the reactive hyperemia phase. All ultrasound images were analyzed using automated edge detection software (v 7.0). FMD was expressed as the percentage change in arterial diameter from baseline to peak hyperemia, calculated using the formula: FMD (%) = [(peak diameter − baseline diameter)/baseline diameter] × 100 [27].

For arteriolar flow-induced dilation (FID), small resistance arterioles were microdissected from adipose tissue biopsies, meticulously cleaned of surrounding connective tissue, and cannulated in a pressurized ex vivo organ chamber, as previously described [27]. Vessels were mounted onto glass microcapillaries using nylon sutures and perfused with Krebs buffer under controlled intraluminal pressure gradients ranging from 10 to 100 cm H_2_O. Real-time vessel diameter measurements were obtained using an inverted Olympus microscope equipped with a video dimension analyzer. To assess endothelial function, arterioles were pre-constricted with endothelin-1 (10^−6^ mol/L) and FID was determined as the percentage increase in vessel diameter at each pressure step relative to the constricted diameter.

### 2.6. Statistical Analysis

Statistical analyses were conducted using SPSS (version 26.0; SPSS Inc., Chicago, IL, USA) and RStudio (version 4.4.1). All assays were performed in triplicate (technical replicates). Continuous variables are reported as mean ± standard deviation, while categorical variables are expressed as frequencies and percentages. Data normality was evaluated using the Shapiro–Wilk test. Based on distribution characteristics, parametric or non-parametric tests were applied: normally distributed variables were analyzed using Student’s *t*-tests or ANOVA with covariate adjustments for age, sex, and race/ethnicity, whereas non-normally distributed data were assessed using the Mann–Whitney U test. The Benjamini–Hochberg false discovery rate (FDR) correction was applied to control for multiple comparisons. Log_2_ fold changes were calculated as the log_2_ ratio of average abundance levels between obese and lean groups. Linear regression models, adjusted for age, sex, and race/ethnicity, were employed to examine associations between proteomic profiles and participant characteristics, with standardized β coefficients indicating the effect per one standard deviation change in the independent variable.

Principal Component Analysis (PCA) was performed using the prcomp function in RStudio on z-score-standardized data (mean = 0, SD = 1), employing Singular Value Decomposition (SVD). Hierarchical clustering was conducted using Ward’s method on similarly scaled data and resulting matrices were visualized using heatmaps to reveal clustering patterns. Data visualization, including bar plots, box plots, and volcano plots, was carried out using the ggplot2 package. Linear relationships between continuous variables were examined using Pearson or Spearman correlation coefficients, as appropriate for the distribution. Finally, protein species with FDR-adjusted *p*-values < 0.05 were submitted to Ingenuity Pathway Analysis (IPA), where biological pathway enrichment was determined using Fisher’s exact test.

Pathway and upstream regulator analyses were conducted using Ingenuity Pathway Analysis (IPA; QIAGEN, Redwood City, CA, USA), a proprietary software platform accessible through the University of Illinois Research Resources Center. IPA pathway annotations are derived through manual curation of published literature describing gene, protein, and metabolic pathways. For each pairwise comparison, proteins showing statistically significant differences in abundance were input into the IPA software for core analysis. Pathway enrichment *p*-values were calculated using Fisher’s Exact Test, followed by false discovery rate (FDR) correction. IPA also computes a Z-score based on the log fold change of input proteins to predict the activation or inhibition status of each pathway. Canonical pathway enrichment results, including statistical values and bar plots, were generated and exported directly from IPA. Additionally, IPA’s upstream regulator analysis feature was used to identify potential transcriptional or signaling regulators responsible for the observed protein expression patterns. These results were exported in tabular format for downstream interpretation.

To identify upstream regulators and associated biological functions, the Downstream Effects and Upstream Regulator modules of IPA were used. Proteins showing statistically significant differences in abundance between comparison groups were uploaded into IPA for core analysis. The Upstream Regulator Analysis predicts potential transcriptional regulators (e.g., cytokines, transcription factors, or complexes) that may explain the observed changes in protein expression. Simultaneously, the Downstream Effects Analysis identifies biological processes and disease functions potentially impacted by these proteins. For each identified relationship, IPA computes a Consistency Score, reflecting the strength and coherence of the data supporting the regulator-function network. A higher consistency score indicates stronger agreement between the observed protein expression patterns and known regulatory pathways. The analysis also reports the Nodal Total (total number of molecules in the network) and the proportion of known regulator-function relationships matched by the input data. The results were exported as summary tables directly from IPA and manually curated to compile the top-scoring networks, which include predicted regulators (e.g., TNF, IL1), affected biological functions (e.g., apoptosis, inflammation, cellular infiltration), and their associated statistical and mechanistic scores.

Predictive modeling was performed using IBM SPSS Statistics (version 29), incorporating both random forest and decision tree algorithms to evaluate the ability of adiposome-derived protein species to predict a spectrum of clinical outcomes. Target variables included obesity, type 2 diabetes, hypertension, dyslipidemia, and hepatic steatosis. Protein concentrations served as continuous predictors in all models. For the random forest approach, models were constructed using 122 samples to predict obesity and 75 samples for cardiometabolic outcomes within the obese subgroup. Forests were grown with 300 to 500 trees, a maximum depth of 20, and inverse frequency-based weighting to address class imbalance. Decision trees were generated using the Classification and Regression Trees (CART) algorithm within SPSS’s Decision Trees module, with parameters set to a minimum parent node size of 5, child node size of 2, and a maximum depth of 3. The Gini index was used as the splitting criterion. All models were pruned and validated using 10-fold cross-validation to minimize overfitting and enhance generalizability. Model performance was assessed based on accuracy, sensitivity, specificity, and area under the receiver operating characteristic curve (AUC), providing a comprehensive evaluation of the predictive utility of adiposomal proteins across diverse cardiometabolic conditions.

## 3. Results

### 3.1. Clinical Characteristics of the Study Participants

All clinical characteristics of the obese (n = 75) and control (n = 47) groups are summarized in Table 1. Age was not significantly different between groups. However, BMI was nearly 89% higher in the obese group, accompanied by substantial increases in total fat percentage (144%) and visceral fat mass (181%) as measured by DEXA (all *p* < 0.01). Obese individuals also demonstrated a significantly elevated resting heart rate (7%, *p* = 0.02), along with higher systolic and diastolic blood pressures, by 11% (*p* < 0.01) and 5% (*p* = 0.02), respectively. Metabolic parameters were consistently more impaired in the obese group, with fasting insulin levels nearly doubled, fasting glucose elevated by 12%, and HOMA-IR more than doubled compared to controls. HbA1c levels were also significantly higher (*p* < 0.01). In terms of lipid profile, total cholesterol and LDL cholesterol were increased by 12% and 16%, respectively, while HDL cholesterol was reduced by 21%. Triglycerides were markedly elevated by 43% in the obese group (all *p* < 0.01).

Liver function was notably altered in the obese group, with albumin decreased by 12% (*p* < 0.01) and alkaline phosphatase elevated by 17% (*p* = 0.003). Although ALT and AST levels trended higher, these differences did not reach statistical significance (*p* = 0.39 and *p* = 0.33, respectively). Inflammatory markers were profoundly elevated in the obese group. IL-6 levels were over fourfold higher, and CRP was nearly sixfold greater than in controls. Leptin concentrations were more than tripled, while adiponectin decreased by 59%, leading to a leptin/adiponectin ratio nearly six times higher in obese individuals. Participants were classified as having high inflammation if their circulating levels of IL6 and CRP were higher than the average in the lean, healthy controls (5.3 pg/mL for IL6 and 0.67 mg/dL for CRP). Systemic inflammation was present in 65.3% of the obese group, compared to 26.1% of controls; all inflammatory markers were higher in the former group (*p* < 0.01). Vascular function was significantly impaired in obesity. In vivo brachial flow-mediated dilation (FMD) was reduced by 67%, while ex vivo arteriolar flow-induced dilation (FID) declined by 37%. Nitric oxide levels were also nearly 49% lower in obese participants (*p* < 0.01), reflecting substantial endothelial dysfunction.

Clinically, the observed metabolic and vascular disturbances manifested as a markedly higher disease burden in the obese group. Hypertension was present in 45.3% of obese individuals, diabetes (defined as HbA1c ≥ 6.5%, fasting glucose ≥ 126 mg/dL, or documented diagnosis in medical records) in 26.7%, insulin resistance (defined as HOMA-IR ≥ 2.6) in 48.0%, dyslipidemia in 45.3%, and hepatic steatosis in 57.3%, none of which were observed in the control group. Obese individuals were classified as having vascular dysfunction if their brachial FMD or arteriolar FID fell below the average values of lean, healthy controls (17.4% for brachial FMD and 76.1% for arteriolar FID). Vascular dysfunction was highly prevalent, with impaired vascular function in 61.3% of obese participants, compared to no impairments in controls. These clinical diagnoses were established through standardized measurements obtained during study visits and corroborated by participants’ medical records.

### 3.2. Adiposome Characteristics

**Figure 1:**
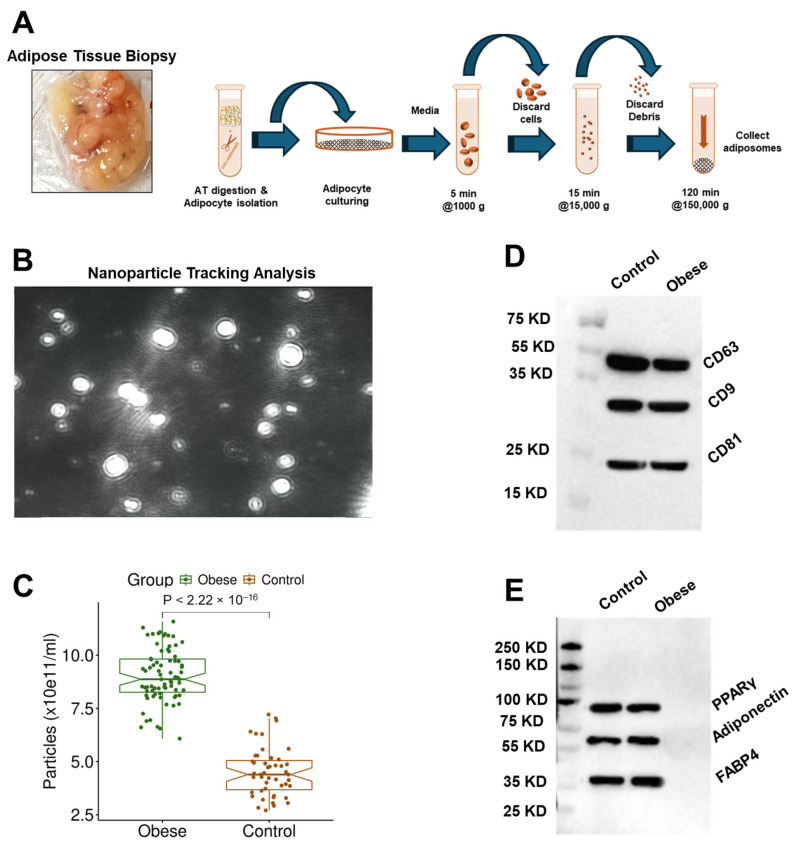
**Isolation and characterization of adiposomes from human visceral adipose tissue (VAT).** (**A**) A representative image of visceral adipose tissue collected during surgery and a schematic of the sequential steps used to isolate adiposomes. (**B**) A representative nanoparticle tracking analysis (NTA) image of isolated vesicles. (**C**) Quantification of adiposome concentration in obese individuals versus lean controls. (**D**) Immunoblot analysis confirms the enrichment of canonical extracellular vesicle markers (CD9, CD81, CD63). (**E**) Immunoblot validation of adipocyte origin through adiponectin, PPARγ, and FABP4 detection in isolated adiposomes.

To examine obesity-associated proteomic alterations in adiposomes and their potential contribution to cardiometabolic dysfunction, adiposomes were isolated from human visceral adipose tissue (VAT) through sequential centrifugation and ultracentrifugation (Figure 1A) and characterized via nanoparticle tracking analysis (NTA, Figure 1B). This analysis confirmed the presence of EVs ranging from 50 to 350 nm in diameter. Quantitative measurements revealed a significantly greater concentration of adiposomes in obese individuals compared to lean controls (9.0 × 10^11^ vs. 4.5 × 10^11^ particles/mL, *p* < 0.001) (Figure 1C). Immunoblotting demonstrated enrichment of canonical EV markers (CD9, CD81, CD63, Figure 1D) and confirmed the absence of lipoprotein contamination (APOB). The presence of PPARγ, adiponectin, and FABP4 (Figure 1E) further validated the adipocyte-derived origin of these vesicles [28]. The presence of tetraspanins and adipocytic proteins was evaluated in over one-third of the total adiposome preparations across all experimental groups. Samples were randomly selected within each group to ensure representative coverage and to assess the consistency of marker expression across biological replicates.

### 3.3. Obesity-Linked Proteomics Shifts

**Figure 2:**
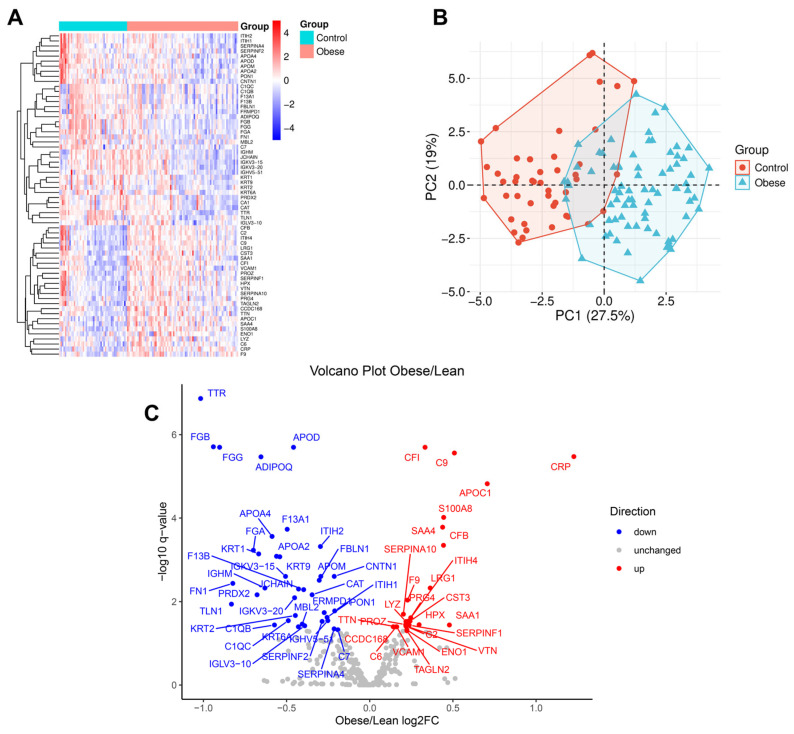
**Differential adiposome proteins in obese and lean individuals**. (**A**) Heatmap of significantly different adiposome proteins across lean and obese groups. (**B**) Principal Component Analysis (PCA) plot of adiposome proteins in obese and lean groups. (**C**) Volcano plot illustrates the differential protein abundance (log_2_ fold change) between obese and lean groups (−log_10_ *p*-value).

To investigate obesity-related alterations in the proteomic profile of VAT adiposomes, differential abundance analysis was conducted on proteins isolated from samples obtained from lean and obese individuals. A comprehensive proteomics approach identified 305 unique proteins, of which 64 showed significant changes (FDR < 0.05). These proteins are visualized in a heatmap (Figure 2A), which reveals clear clustering by group, reflecting consistent obesity-associated proteomic differences. Principal Component Analysis (PCA) further demonstrated a distinct separation between lean and obese groups, with the first two principal components accounting for 27.5% and 19% of the total variance, respectively (Figure 2B), supporting the presence of group-specific proteomic signatures. The volcano plot (Figure 2C) highlights the significantly different proteins, including 3 that were upregulated by more than 1.4-fold (log_2_FC > 0.5) and 15 that were downregulated by more than 1.4-fold (log_2_FC < −0.5) in obese individuals. The remaining 46 proteins displayed biologically modest yet statistically significant differences (|log_2_FC| < 0.5). Importantly, no significant differences in proteomic profiles were observed based on participants’ sex or racial/ethnic background.

**Figure 3:**
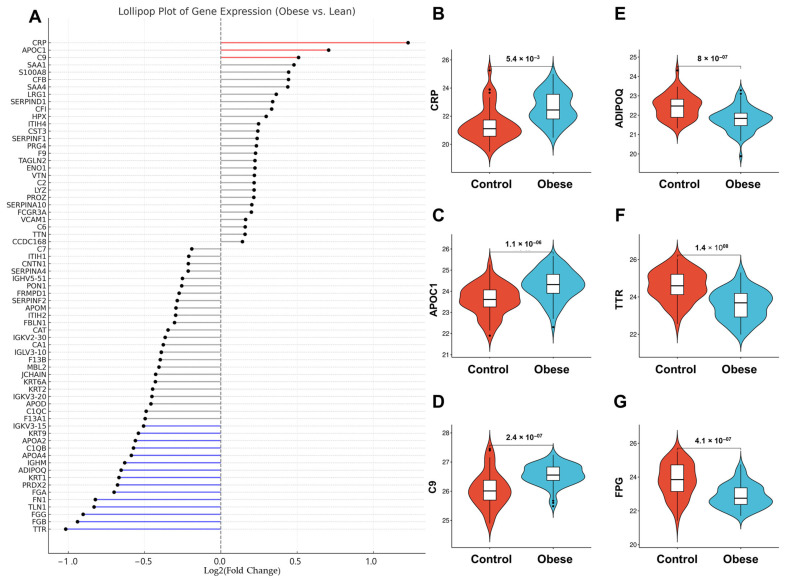
**Differential protein profiles in adiposomes of obese and lean individuals**. (**A**) Lollipop plot for proteins with statistically significant changes (FDR value < 0.05) stratified by biological significance (log_2_FC). Red: Upregulated (log_2_FC > 0.5, FDR < 0.05), Blue: Downregulated (log_2_FC < −0.5, FDR < 0.05), Gray: Not biologically significant. (**B**–**G**) Violin plots showing examples of the significantly altered proteins between the obese and lean groups.

To further resolve and rank the abundance patterns of significantly altered proteins, a lollipop plot was generated (Figure 3A) displaying log_2_ fold changes (FDR < 0.05), stratified by both the direction and magnitude of change. Among the upregulated proteins, C-reactive protein (CRP; Figure 3B) showed the greatest increase in obese individuals (fold change = 2.23), consistent with its established role as an inflammatory biomarker associated with metabolic dysfunction and cardiovascular disease. Apolipoprotein C-1 (APOC1; Figure 3C) and Complement C9 (C9; Figure 3D) were also significantly elevated (fold change = 1.94 and 1.84, respectively), indicating potential alterations in lipid metabolism and immune response within obese adipose tissue.

Conversely, adiponectin (ADIPOQ; Figure 3E), an insulin-sensitizing hormone secreted by adipocytes, was modestly but significantly downregulated (log_2_FC = −0.52). Multiple structural and metabolic proteins also exhibited marked suppression in obesity. Transthyretin (TTR; Figure 3F), a carrier protein for thyroxine and retinol-binding protein, showed the strongest downregulation (log_2_FC = −1.02). The three fibrinogen subunits, Fibrinogen beta chain (FGB; Figure 3G, log_2_FC = −0.94), gamma chain (FGG, log_2_FC = −0.90), and alpha chain (FGA, log_2_FC = −0.67), were consistently reduced, suggesting coordinated suppression of the fibrinogen complex. Additionally, Fibronectin 1 (FN1), a key extracellular matrix glycoprotein (log_2_FC = −0.78), and Talin-1 (TLN1), a cytoskeletal adapter essential for integrin activation and focal adhesion (log_2_FC = −0.63), were significantly downregulated, indicating impaired tissue structure and cellular adhesion dynamics in obese VAT.

Furthermore, several apolipoproteins exhibited a consistent trend of downregulation. Apolipoprotein A4 (APOA4) and Apolipoprotein C-II (APOC2) were notably reduced, with log_2_FC values of −0.63 and −0.59, respectively. Apolipoprotein C-III (APOC3) (log_2_FC = −0.51) and Apolipoprotein A-II (APOA2) (log_2_FC = −0.52) were also significantly decreased, reflecting coordinated alterations in plasma lipid transport and regulatory pathways. In parallel, Peroxiredoxin-2 (PRDX2), a redox-active protein with antioxidant functions, was downregulated (log_2_FC = −0.58), suggesting perturbations in oxidative stress response mechanisms. Immune-related Proteins also demonstrated reduced abundance. Immunoglobulin heavy constant mu (IGHM) and Immunoglobulin kappa variable 3-15 (IGKV3-15) each showed a log_2_FC of −0.56, indicating suppression of immune signaling components. Additionally, KRT1, a type II keratin essential for epidermal integrity, was downregulated (log_2_FC = −0.67), as was KRT9 (log_2_FC = −0.54), a palmoplantar-specific keratin essential for mechanical resilience, pointing to possible disruptions in tissue structural maintenance.

### 3.4. Proteomic Shifts in VAT-Derived Adiposomes Mirror Cardiometabolic Risk Profiles in Obesity

To contextualize the observed proteomic shifts, we conducted multivariable linear regression analyses examining the associations between clinical and demographic variables and protein abundance in VAT-derived adiposomes from obese individuals. All models were adjusted for the complete set of covariates.

**Figure 4:**
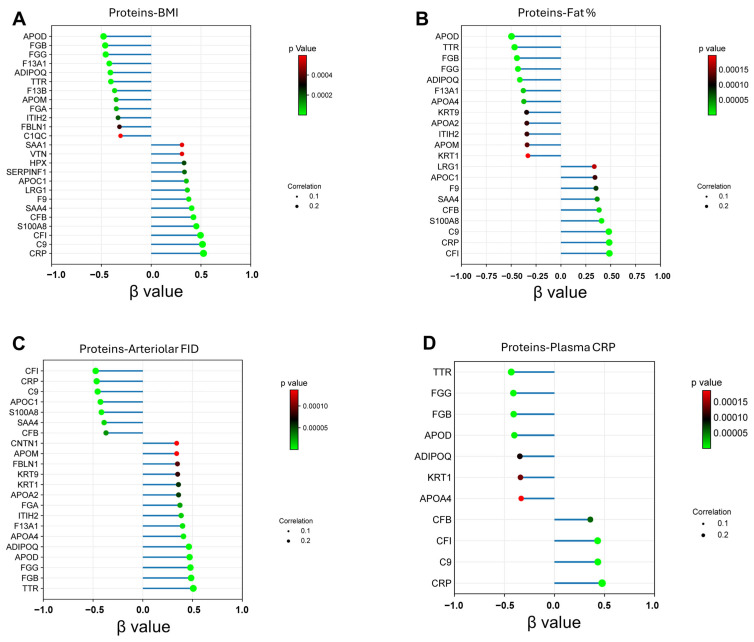
**Regression analysis and association of adiposome lipids with obesity status**. (**A**) Regression plot showing the association between lipid species and BMI (**A**), fat percentage (**B**), arteriolar FID (**C**), and plasma CRP (**D**), arranged by their *β*-values (linear regression) and their associated corrected *p* values. The size of each lollipop head reflects the strength of the correlation coefficient. The reference circles labeled “Correlation 0.1” and “Correlation 0.2” illustrate the relative scale used for head sizes in the plots.

Twelve adiposome proteins exhibited positive *β* coefficients, indicating a direct correlation with BMI. In comparison, thirteen proteins showed negative *β* values, reflecting an inverse association with BMI (Figure 4A). Significant associations were also observed between adiposome proteins and clinical metrics such as body fat percentage measured by DEXA (Figure 4B), ex vivo arteriolar flow-induced dilation (FID; Figure 4C), and circulating CRP levels (Figure 4D). Notably, positive correlations were identified between nitric oxide levels and proteins, including TTR, APOD, and ADIPOQ. In contrast, inverse relationships were found between adiposome CRP and circulating adiponectin, as well as between adiposome TTR and fasting insulin levels.

Examining these proteomic shifts in greater detail revealed a consistent pattern of associations. Among the proteins upregulated in obesity, CRP, C9, and APOC1 not only showed elevated abundance but also demonstrated strong, consistent links across metabolic, vascular, and inflammatory domains. These proteins formed a converging signature: both CRP and C9 were associated with visceral fat expansion, hormonal dysregulation (elevated leptin), and impaired endothelial function, hallmarks of cardiometabolic stress. While APOC1 showed weaker vascular associations, it paralleled the inflammatory signature through its strong correlation with leptin, suggesting a role in adipose tissue remodeling. Together, these interconnected profiles hint at a coordinated molecular response underlying obesity’s systemic impact.

Complementing these findings, a parallel pattern was observed among several downregulated adiposome proteins, which appeared to play protective roles. The reduced abundance of proteins such as TTR, ADIPOQ, FGB, and FGG was consistently associated with adverse clinical features. These proteins showed negative associations with visceral fat, leptin, the leptin/adiponectin ratio, and circulating IL-6 levels while exhibiting positive correlations with brachial artery flow-mediated dilation, a marker of vascular health. The directionality of these associations suggests that their downregulation in obesity may contribute directly to endothelial dysfunction, immune dysregulation, and elevated leptin levels. Additional details are provided in Supplementary Table S1.

### 3.5. Network-Level Analysis of Obesity-Associated Molecular Dysregulation

Protein network analysis was performed using Ingenuity Pathway Analysis (IPA), where differentially abundant proteins (FDR < 0.05) were mapped onto canonical interaction networks. Pathway enrichment scores were derived using Fisher’s exact test, while consistency scores were calculated based on observed-to-expected overlaps between dataset molecules and known pathway components. Network analysis offered a system-level perspective on the proteomic alterations identified. The Adipokine-Amyloid-Oxidative Stress Network, enriched with altered ADIPOQ, APOA4, CRP, and TTR, closely reflected the systemic insulin resistance, inflammation, and oxidative stress previously described. Similarly, the Complement Activation Network, characterized by increased C9 and CRP and decreased C1QB, aligned with the chronic low-grade inflammation observed in obesity. The TLR-MyD88-Coagulation Network, which included downregulated FGA, FGG, and APOA2, provided further insight into vascular remodeling and metabolic dysfunction. Structural disruptions were captured in the Cytoskeleton-Adhesion Network, involving reduced abundance of KRT1 and KRT9, while the RAF-MAPK Pathway encompassed redox and extracellular matrix-related proteins such as FN1, TLN1, and PRDX2. Together, these network-level associations reinforce prior clinical observations, underscoring that obesity involves coordinated dysregulation across inflammatory, structural, and metabolic pathways. A complete network analysis is provided in Supplementary Table S2.

### 3.6. Upstream Regulators and Canonical Pathways Associated with Adiposome Proteomic Shift

To comprehensively characterize upstream regulatory influences underlying obesity-related molecular alterations, we employed two complementary analytical approaches: a regulator-function table and a z-score heatmap. The regulator-function table (Table 2) maps upstream regulators to pathophysiological processes such as inflammation, apoptosis, and immune activation based on known gene-disease relationships and consistency scores. This analysis provides insight into how dysregulated genes contribute to clinical phenotypes.

**Figure 5:**
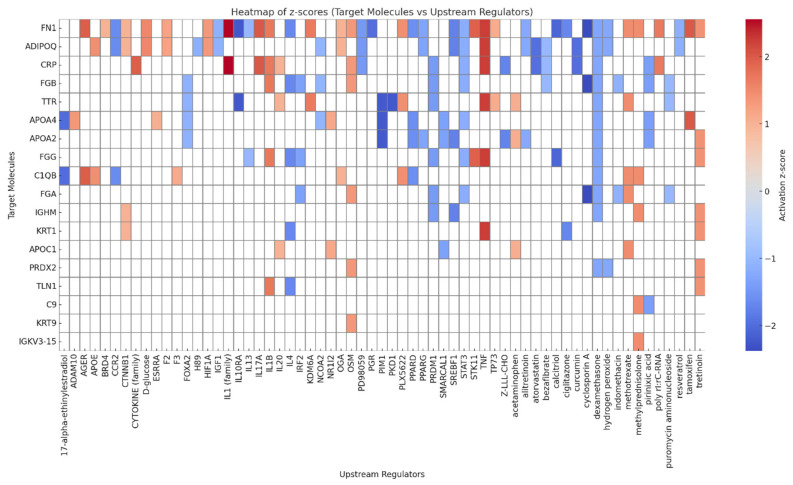
Heatmap of *z*-scores (target molecules vs. upstream regulators).

In parallel, the z-score heatmap (Figure 5) quantifies activation or inhibition patterns of upstream regulators using z-scores derived from the abundance of their downstream targets, offering a high-resolution view of transcriptional dynamics at the gene level.

Upstream analysis identified TNF and IL1 as major pro-inflammatory regulators, with z-scores of +2.24 and +2.56, respectively. These cytokines influenced a broad set of differentially abundant proteins. TNF was associated with the upregulation of CRP and S100A8, as well as the suppression of ADIPOQ, FN1, and FGG. Similarly, IL1 promoted CRP upregulation and showed mixed regulatory effects on targets such as FN1. Activation of the complement complex (CG, z = +2.00) further highlighted an amplified innate immune response. In contrast, calcitriol and synthetic estrogens (e.g., 17α-ethinylestradiol) were predicted to be inhibited, affecting downregulated proteins such as FN1, APOA4, and FGG, all of which were suppressed in the obese adiposome.

**Figure 6:**
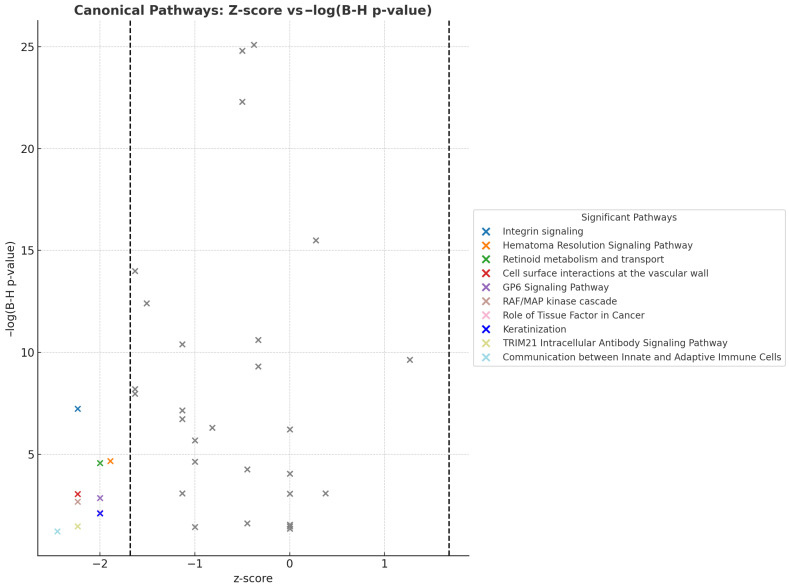
**Canonical pathways; *z*-score vs. −log (B-H *p*-value).** Colored crosses denote significant pathways meeting both the −log(B-H *p*-value) and |*z*-score| thresholds (here, |*z*-score| ≥ 1.96). Grey crosses indicate pathways that did not meet the *z*-score threshold while meeting the −log(B-H *p*-value) threshold and are included for reference. Vertical dashed lines mark the ±1.96 *z*-score cut-offs, separating potentially activated (positive *z*-score) and inhibited (negative *z*-score) pathways. The legend lists only the significant colored pathways for clarity. Note: The “Role of Tissue Factor in Cancer” and “Keratinization” pathways exhibited identical z-scores (–2.0) and nearly identical –log(B–H *p*-values) (2.14 and 2.10, respectively). Consequently, their data points are closely clustered on the plot and may initially appear as a single point.

At the pathway level, canonical signaling through integrins, GP6, RAF/MAPK, and retinoid transport was significantly inhibited (*z* < −1.645, *p* < 0.05), aligning with earlier findings of suppressed extracellular matrix (ECM) proteins and vascular dysfunction. Notably, the suppression of the retinoid receptor pathway corresponded with the marked downregulation of TTR. While the Complement Cascade and Coagulation System were strongly enriched (−log(*p*) > 10), they exhibited borderline or inconsistent inhibition (z ≈ −1.6), potentially reflecting dysregulated or unresolved immune responses. The most suppressed pathway, linking innate and adaptive immune cells, had a z-score of −2.449, suggesting a broad impairment in coordinated immune surveillance. This may contribute to the chronic, unresolved inflammation characteristic of obesity, potentially explaining the mixed directional trends observed among immune markers (Figure 6).

### 3.7. Adiposome Protein Signatures as Predictors of Cardiometabolic Diseases

**Figure 7:**
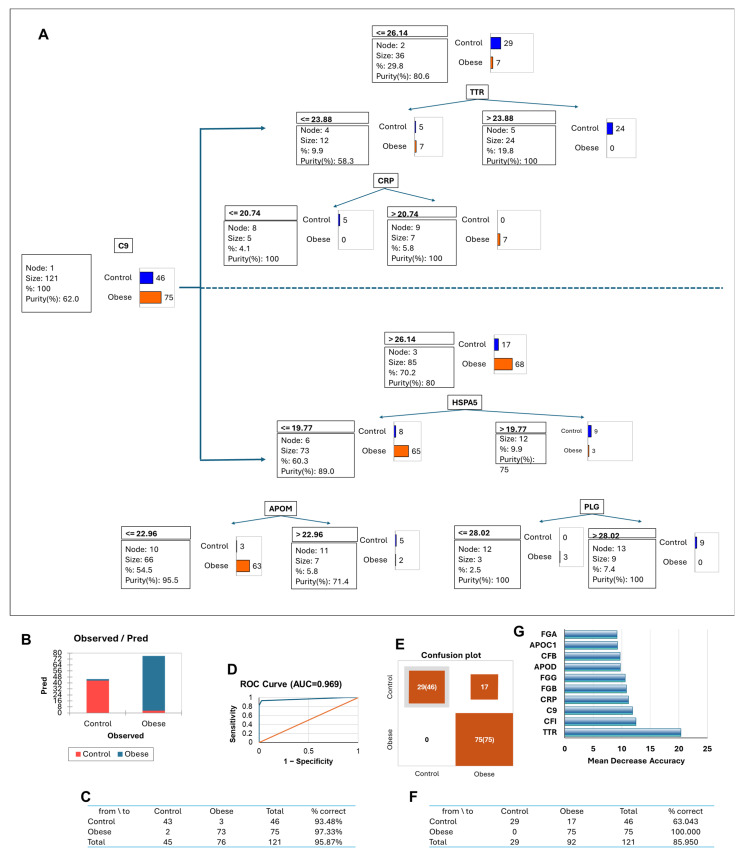
**Predictive modelling of obesity status using adiposome proteomic profiles**. (**A**) A decision tree classification model constructed using differentially abundant adiposome proteins, showing a hierarchical decision path. (**B**,**C**) Bar plot and table showing model accuracy in the control and obese groups. (**D**) Receiver Operating Characteristic (ROC) curve for the decision tree model, yielding an AUC = 0.969. (**E**,**F**) Confusion matrix and table illustrating correct and incorrect classifications. (**G**) Variable importance plot from the random forest model.

To explore the predictive value of adiposome proteomic profiles, we developed supervised machine learning models, specifically decision tree and random forest approaches, to classify cardiometabolic conditions among obese individuals. These included diabetes, hypertension, dyslipidemia, and hepatic steatosis. As a first step, models were applied to distinguish between the control and obese groups based on adiposome protein features. The decision tree model achieved ~96% classification accuracy, with C9 (complement component 9) serving as the root node and primary classifier. Additional contributing proteins included TTR, CRP, HSPA5, APOM, and PLG (Figure 7A). Performance was slightly higher in the obese group, with an AUC of 0.969, indicating excellent discrimination (Figure 7B–D). The random forest confusion matrix showed 100% correct classification of obese individuals and 63% of controls (Figure 7E,F). Notably, C9, TTR, CRP, and FGB emerged as top predictors (Figure 7G).

When hypertension was the endpoint, the Decision-tree model achieved an overall accuracy of 92%, correctly identifying every hypertensive individual (sensitivity = 100%) and 85% of normotensive controls (specificity), with an AUC of 0.938 (Figure S1A–C). The random forest model, on the other hand, predicted the non-hypertensive with more accuracy (68%) than the hypertensive (38%), with the most influential proteins being SERPINA10, ALDOB, CPN2, SERPINA6, and IL1RAP (Figure S1D,E).

A parallel analysis targeting diabetes produced stronger performance, reaching 97% overall accuracy and an AUC of 0.991; only two of seventy-five subjects were misclassified using the Decision-tree model (Figure S1F–H). The random forest, on the other hand, predicted both the diabetic and non-diabetic with a lower accuracy but still identified important features such as MMP2, CFHR4, S100A9, CRP, and SERPINA1 (Figure S1I,J). The predictive models built on adiposome proteomic profiles also performed well in classifying individuals with dyslipidemia and hepatic steatosis. For hepatic steatosis, the decision tree model achieved a high area under the curve (AUC) of 0.939 with an overall classification accuracy of approximately 92% (Figure S2A–C), while the random forest model showed a more modest accuracy of 60% (Figure S2D). Key proteins contributing to the prediction included LYEV1, S100A9, GAPDH, and CLEC38, many of which are associated with inflammation and coagulation pathways (Figure S2E). Similarly, for dyslipidemia, the decision tree model achieved an AUC of 0.908 with an overall accuracy of approximately 88% (Figure S2F–H), whereas the random forest model reached 61% accuracy (Figure S2I). The top predictive proteins for hepatic steatosis included TNC, GAPDH, CA2, and C9, underscoring the role of inflammatory mediators and lipid-handling proteins in this condition (Figure S2J).

## 4. Discussion

This study provides novel evidence that visceral adipose tissue-derived adiposomes reflect and potentially contribute to the systemic metabolic derangements associated with obesity. Through high-resolution proteomic analysis, we observe that the molecular composition of adiposomes is substantially altered in obesity, aligning closely with clinically observed phenotypes of inflammation, insulin resistance, vascular dysfunction, and dyslipidemia. These findings support the hypothesis that adiposomes are not only byproducts of adipocyte metabolism but may represent biologically active cargos disseminating pathological signals across the body.

The proteomic alterations observed in adiposomes from obese individuals reveal a robust and coordinated molecular signature of chronic low-grade inflammation and immune dysregulation. Notably, the upregulation of C-reactive protein (CRP), complement component C9, and apolipoprotein C-I (APOC1) stands out as a hallmark of this inflammatory response. CRP and C9 are well-established mediators of innate immune activation and systemic inflammation, respectively [29,30]. Their enrichment in adiposomes suggests that these vesicles may actively propagate inflammatory signals throughout the body, either by delivering immune-stimulating cues to recipient cells or by serving as carriers of pro-inflammatory mediators that sustain immune activation [31]. The strong correlations between these proteins and visceral adiposity, elevated leptin, and impaired vascular function reinforce the link between adiposome cargo and systemic metabolic and vascular disturbances in obesity. For example, adipocyte-derived exosomes from obese mice were shown to induce insulin resistance and elevate pro-inflammatory cytokine release in lean mice [32]. This suggests that these proteins may actively drive obesity-related pathophysiology rather than merely reflect it.

Building on this concept, our previous study showed that adiposomes derived from obese individuals and type 2 diabetes significantly impaired endothelial nitric oxide (NO) production compared to those from lean, healthy controls [12]. Instead of supporting vascular health, these adiposomes promoted oxidative stress and peroxynitrite formation, disrupting shear stress responses, albumin uptake, and flow-induced dilation in healthy arterioles. These findings highlight a clear role for adiposome-derived factors in reducing NO bioavailability and compromising endothelial function. This is echoed by findings that circulating vesicles from obese humans provoke endothelial inflammation and reduce NO bioavailability in target cells compared to vesicles from lean individuals [33].

In the current study, we observed that NO levels positively correlate with transthyretin (TTR), apolipoprotein D (APOD), and adiponectin (ADIPOQ), proteins that were significantly downregulated in obese adiposomes. This suggests that the diminished presence of these protective proteins may be mechanistically linked to NO deficiency and vascular dysfunction in obesity. Adiponectin, in particular, is well-established for enhancing insulin sensitivity and has been shown to upregulate NO production in vascular endothelial cells [34]. Adiponectin also acts as a potent vasodilator in both rat and mouse vessels, directly relaxing isolated aortic and mesenteric artery rings. This effect is mediated by the opening of voltage-gated potassium (Kv) channels [35]. Furthermore, chronic administration of adiponectin restores NO as the primary mediator of flow-induced dilation (FID) in human resistance arterioles. This restoration depends on the adiponectin receptor 1 (AdipoR1); silencing or inhibiting AdipoR1 blocks the effect, confirming its critical signaling role in microvascular NO production and endothelial function [36]. Therefore, it is possible that the observed depletion of adiponectin from adiposomes may limit systemic bioavailability or disrupt its delivery to endothelial cells [37,38], thereby contributing to the endothelial dysfunction characteristic of obese individuals. While the current study highlights a potential mechanistic pathway, future mechanistic investigations are needed to directly test the causal role of these proteins in regulating NO signaling and vascular function.

In support of this mechanistic link, we identified a consistent downregulation of several proteins with well-established protective roles in adiposomes from obese individuals, most notably transthyretin (TTR). TTR, a tetrameric carrier of thyroxine (T4) and retinol-binding protein 4 (RBP4), has been increasingly recognized for functions extending beyond endocrine transport, including the regulation of metabolic and vascular homeostasis [39]. Positioned at the metabolism and cardiovascular health crossroads, TTR plays a pivotal role in maintaining systemic balance [40], and in our study, it exhibited the strongest downregulation among the identified adiposome-associated proteins in obesity. Notably, clinical proteomic studies have also reported lower transthyretin levels in obese and diabetic patients compared to controls. Thus, the marked loss of TTR in obese adiposomes mirrors its systemic decline in metabolic disease, potentially exacerbating the disruption of metabolic and vascular homeostasis [41].

In the liver, TTR plays a key role in glucose homeostasis by modulating glucose transporter levels (GLUT1, GLUT3, GLUT4), enhancing pyruvate kinase M (PKM) activity, and reducing mitochondrial oxidative stress [42], mechanisms vital for metabolic flexibility under nutrient excess. Its function in thyroxine transport ensures proper thyroid hormone distribution, a major regulator of energy metabolism and thermogenesis, both often impaired in obesity [43]. By stabilizing and transporting retinol-bound RBP4, TTR also supports vitamin A delivery, which is essential for immune function and lipid metabolism [44]. TTR further contributes to immune balance by promoting myeloid cell differentiation and modulating chemokine signaling, helping to regulate innate immunity and limit chronic inflammation, a hallmark of obesity [39]. These diverse functions highlight TTR as a molecular safeguard across systems commonly disrupted in obesity; yet the extent to which TTR exerts similar functions in adipose or vascular compartments remains to be fully elucidated. Although TTR is primarily synthesized in the liver and choroid plexus, recent transcriptomic [45] and experimental studies [46,47] have demonstrated its expression at low levels in adipose tissue, suggesting potential local production in addition to systemic uptake. The presence of TTR in adiposomes observed in this study may reflect such local synthesis or selective packaging from circulating sources, aligning with its emerging role in metabolic tissue crosstalk. In the current study, adiposome levels of TTR demonstrated inverse correlations with visceral adiposity, systemic inflammation, and endothelial dysfunction, as well as a strong direct correlation with flow-mediated vasodilation. Overall, these associations, along with the marked reduction in TTR in obese adiposomes, may signal the loss of a key regulator of metabolic, vascular, and immune homeostasis, positioning TTR as a promising biomarker and therapeutic target in obesity.

A particularly compelling aspect of our findings is the coordinated downregulation of the fibrinogen complex, comprising the alpha (FGA), beta (FGB), and gamma (FGG) chains, in adiposomes derived from obese individuals. Beyond its classical role in coagulation, fibrinogen contributes to vascular homeostasis by modulating endothelial barrier integrity, mediating leukocyte recruitment, and facilitating tissue repair [48]. The depletion of fibrinogen subunits in adiposome cargo may, therefore, signify a loss of vesicle-mediated vascular support, potentially contributing to endothelial dysfunction and increased vascular permeability characterizing obesity [49]. Fibrinogen has likewise been identified as a cargo by itself in circulating EVs in other inflammatory conditions; therefore, its diminished inclusion in obese adiposomes may weaken vesicle-mediated support of normal endothelial repair and anti-inflammatory signaling, contributing to the microvascular fragility of obesity [50]. In our dataset, FGB and FGG were not only reduced in obese adiposomes but also positively associated with vascular function (e.g., brachial FMD) and inversely correlated with IL-6, a key inflammatory cytokine. Furthermore, this reduction coincided with the downregulation of structural matrix proteins such as fibronectin and talin-1, indicating broader extracellular matrix (ECM) disintegration and diminished regenerative capacity within the obese adipose microenvironment [51]. Together, these findings raise the possibility that altered adiposome-mediated transport of fibrinogen and ECM components may contribute to the progression of microvascular damage and inflammation in obesity.

Similar to TTR and fibrinogen, apolipoprotein D (APOD) was significantly downregulated in adiposomes derived from obese individuals and demonstrated inverse correlations with key metabolic and inflammatory dysregulation indicators, including IL-6 and the leptin/adiponectin ratio. APOD is well-recognized for its involvement in lipid transport, high-density lipoprotein (HDL) remodeling, and antioxidative defense mechanisms [52,53]. Its reduced abundance in the current study mirrors observations from atherosclerosis models, where APOD has been shown to attenuate inflammatory responses and oxidative stress [54]. These findings suggest that adiposome-associated APOD may play a protective role in the obese adipose environment by modulating inflammation and preserving endothelial function. This interpretation aligns with studies reporting that the upregulation of APOD is associated with reduced oxidative stress and inflammation in various models. Accordingly, the downregulation of APOD in obese adiposomes represents a failure to deploy this protective, antioxidative response via vesicles in the obese state [52].

Adding further to this pattern of disrupted protective signaling, we observed significant downregulation of peroxiredoxin-2 (PRDX2), an antioxidant enzyme involved in redox regulation and cellular defense against oxidative stress [55]. While PRDX2 has traditionally been associated with redox homeostasis, its role in metabolic regulation appears to be context-dependent. Although some studies suggest that PRDX2 deficiency can paradoxically enhance insulin signaling and glucose uptake via altered protein tyrosine phosphatase [56], others have shown increased PRDX2 abundance following exercise, particularly in erythrocytes from obese individuals with type 2 diabetes, where it may act as a compensatory antioxidant mechanism to restore redox balance and improve insulin responsiveness [57]. In the context of obesity-related oxidative stress, such antioxidant responses may be especially critical. The loss of PRDX2 in obese adiposomes may, therefore, exacerbate oxidative damage and impair metabolic homeostasis. Together, the depletion of APOD and PRDX2 highlights a broader impairment in the antioxidative and anti-inflammatory functions of adiposome cargo in obesity. These proteomic alterations may contribute to a local adipose tissue environment that is more susceptible to inflammation, oxidative stress, and insulin resistance, which are central to the pathogenesis of obesity-related vascular and metabolic complications.

In contrast to the downregulation of protective proteins, several pro-inflammatory mediators were significantly upregulated in obese adiposomes, suggesting a pathogenic remodeling of vesicular content that may actively propagate systemic inflammation and vascular dysfunction. Among these, C-reactive protein (CRP) and complement component 9 (C9) were most prominent, exhibiting strong positive correlations with visceral adiposity and leptin dysregulation, along with inverse associations with brachial FMD. CRP, a well-established inflammatory marker, has been shown to activate NF-κB signaling, promote cytokine release, and contribute to insulin resistance [58]. Its presence within adiposomes may facilitate the vesicular dissemination of these pro-inflammatory effects, extending their reach beyond local tissue sites. Similarly, C9, a terminal component of the membrane attack complex, showed one of the strongest inverse relationships with endothelial function, reinforcing its proposed role in promoting endothelial injury through complement activation. These findings are consistent with reports implicating C9 in vascular damage in sepsis and atherosclerosis models [59,60,61], and they suggest that C9-enriched adiposomes may represent a previously underappreciated mechanism of complement-mediated vascular dysfunction in obesity.

Collectively, the reciprocal shift from protective proteins like TTR, APOD, and PRDX2 to pro-inflammatory mediators such as CRP and C9 highlights a coordinated reprogramming of adiposome cargo in obesity. This proteomic remodeling mirrors the underlying inflammatory and metabolic state and may serve as a mechanistic conduit through which adipose tissue influences systemic vascular and metabolic homeostasis. The enrichment of inflammatory and complement proteins within adiposomes suggests these vesicles may actively disseminate pro-inflammatory signals to distal tissues, thereby exacerbating insulin resistance and endothelial dysfunction. Supporting this paradigm, proteomic analysis of plasma exosomes in obese-diabetic patients revealed a similar shift. For instance, complement C9 was elevated while antioxidant proteins like hemopexin were reduced, paralleling the pro-inflammatory cargo enrichment seen in our adiposome profiles [62].

The phenotypic reflections of these proteomic alterations are further supported by upstream regulator analysis, which identified TNF-α and IL-1 as dominant drivers of the observed patterns. These cytokines are well-established in obesity-induced inflammation and have been shown to impair insulin signaling [63], promote endothelial activation [64], and contribute to adipocyte dysfunction [65]. The prominence of TNF as a central regulator of adiposome proteome composition raises the intriguing possibility that vesicle cargo may serve not only as a readout of inflammatory signaling but also as a diagnostic or therapeutic target in metabolic disease. These findings collectively underscore the utility of adiposome profiling in identifying mechanistic biomarkers of obesity-related complications and highlight new avenues for targeted intervention to disrupt inflammatory signaling cascades at the vesicle level.

These mechanistic insights into adiposome cargo reprogramming raise essential questions about their translational utility, specifically, whether proteomic alterations in adiposomes can be leveraged to predict clinical outcomes. To address this, we employed supervised machine learning models to evaluate the diagnostic and classification potential of adiposome protein signatures across obesity-related cardiometabolic conditions. The strong predictive performance of decision tree and random forest models in classifying cardiometabolic conditions highlights the potential of adiposome proteomic signatures for disease risk stratification in obesity. However, since adipose tissue sampling is inherently invasive and typically performed during surgery, the use of such signatures as routine screening biomarkers is limited. Future studies should assess whether similar proteomic patterns can be detected in more accessible samples, such as circulating adiposomes or exosomes. Our models achieved high classification accuracies for obesity (96%), diabetes (97%), hypertension (92%), hepatic steatosis (92%), and dyslipidemia (88%), with AUCs consistently exceeding 0.90 in decision tree approaches. These findings align with emerging evidence that adiposome cargo reflects the pathological state of adipose tissue and can act as a sentinel of metabolic dysfunction [66,67]. Key predictive proteins such as C9, TTR, CRP, and S100A9 are not only differentially abundant in obesity but also implicated in the pathogenesis of endothelial dysfunction, systemic inflammation, and insulin resistance [61,68,69,70], further validating their mechanistic relevance.

Importantly, several proteins identified by the models have well-documented biological functions that mirror their predictive roles. For instance, complement component C9, the root classifier in the obesity model, is a terminal effector of the membrane attack complex and has been implicated in metabolic inflammation and vascular injury [59]. CRP, a consistent top predictor across multiple conditions, is known for its role in low-grade chronic inflammation associated with insulin resistance and cardiovascular disease [71]. The emergence of matrix metalloproteinases (e.g., MMP2) and S100A9 in diabetes and steatosis prediction models further supports the role of adiposome-mediated remodeling and inflammatory signaling in these diseases [72]. Additionally, proteins such as the SERPIN family and IL1RAP, identified in the hypertension model, link adiposome cargo to coagulation and interleukin signaling, two key pathways known to be dysregulated in obesity-related hypertension [73,74,75]. Collectively, these results position adiposome proteomics as a promising platform for machine learning-driven disease prediction, with potential applications in early diagnosis, patient stratification, and precision therapeutics in metabolic disease management.

It is worth mentioning that several proteins identified in adiposomes are not traditionally associated with adipocyte expression, raising the possibility of selective cargo loading from surrounding cell types or systemic sources. One prominent example is S100A9, a protein primarily produced by neutrophils and macrophages with well-established roles in inflammation and vascular pathology [76]. Although not a classical adipokine, S100A9 is inducible in adipose tissue during inflammatory states, and mouse models lacking S100A9 exhibit reduced adipose neutrophil infiltration and altered lipolysis under high-fat diet conditions [77]. These findings suggest that such proteins, though not adipocyte-specific, may be selectively incorporated into adiposomes in response to metabolic stress, reflecting immune-metabolic crosstalk. Supporting this concept, proteomic studies and recent literature have shown that adipose-derived EVs carry proteins not highly expressed in adipocytes themselves, indicating contributions from immune and stromal vascular fraction cells, or even uptake from distal tissues [22,78,79]. Notably, vesicle-mediated transfer of caveolin-1 from endothelial cells to adipocyte-derived EVs, despite genetic deletion of caveolin-1 in adipocytes, provides direct evidence for intercellular protein acquisition within adipose tissue [80]. These findings highlight the dynamic and integrative nature of adiposome cargo composition, which is influenced by nutritional and metabolic status (e.g., obesity, fasting), and support the role of adipose EVs as mediators of both intra-tissue and systemic communication under stress conditions.

While human data on adipose-derived EVs remain limited, several rodent and preclinical studies have provided valuable insights into their proteomic composition and therapeutic potential. Recent proteomic analyses of mesenchymal stem/stromal cell (MSC)-derived EVs have highlighted their complex and selective protein cargo. For instance, exosomes from mouse adipose-derived MSCs (ADSCs) contained 1185 proteins, and were enriched in pathways involved in metabolism, cytoskeletal remodeling, and tissue regeneration (MAPK, VEGF, Jak-STAT) [21]. Similarly, porcine ADSC-derived EVs harbored 4937 proteins, with selective enrichment in angiogenesis, coagulation, inflammation, and extracellular matrix remodeling, key processes in tissue repair [22].

Several studies have also highlighted the relevance of EV protein cargo in driving regenerative and immunomodulatory processes. Wang et al. [23] reported that adipose-derived EVs are particularly enriched in proteins associated with immune regulation and antioxidant activity. Complementing these findings, Lee et al. [24] demonstrated that adipose tissue-derived mesenchymal stem cells can reproducibly generate exosomes that carry antioxidant enzymes such as PRDX 1, 2, 4, and 6, contributing to renal protection in animal models of acute kidney injury. Niada et al. [21] further showed that conditioned media and EVs derived from human adipose stromal cells (ASCs) were enriched in factors involved in extracellular matrix remodeling and beta-cell proliferation. Extending beyond culture media from isolated cell models, Watanabe et al. [25] profiled EVs directly from mouse skeletal muscle, heart, liver, and adipose tissue, identifying tissue-specific markers and proteomic signatures. Adipose-derived EVs showed the highest accessibility to circulation, highlighting their role in systemic interorgan communication. Collectively, these findings highlight the functional significance of adipose-derived EVs as potent mediators of systemic regeneration, immune modulation, and interorgan communication. Building on this growing body of work, our study provides the first comprehensive proteomic characterization of visceral adipose tissue-derived adiposomes in humans, uncovering a broader spectrum of functional proteins, including TTR, fibrinogen subunits, and S100A9, not previously identified in this context. By linking specific alterations in adiposome protein cargo to clinical markers of inflammation, endothelial dysfunction, and cardiometabolic risk, our findings underscore the unique value of human tissue-based EV profiling in revealing novel mediators of obesity-related disease.

While our study provides comprehensive insights into the proteomic alterations of adiposomes in obesity and their associations with clinical cardiometabolic outcomes, several limitations should be acknowledged. First, although the sample size was sufficient to detect significant differences and build robust predictive models, the cohort was cross-sectional in nature. This limits causal inference and prevents definitive conclusions about whether observed adiposome proteomic changes are drivers or consequences of metabolic dysfunction. Longitudinal studies are needed to determine the temporal relationship between adiposome cargo alterations and disease progression or therapeutic response. Second, while machine learning models yielded high classification accuracy for several cardiometabolic outcomes, external validation in independent and more diverse cohorts is required to assess generalizability. Factors such as age, sex, ethnicity, and comorbidities may influence adiposome composition and should be explored in future studies. Third, although proteomic profiling offers profound insight into cargo composition, it does not capture post-translational modifications, protein activity states, or interactions with recipient cells. Functional studies are needed to directly test the biological effects of specific adiposome proteins, particularly those identified as predictive or mechanistically relevant, in target tissues such as the endothelium, liver, and skeletal muscle. A key limitation of this study is the inability to resolve proteoforms. The bottom-up proteomics approach used here allows for robust identification of proteins and some modified peptides; however, it does not capture the full structural and functional diversity of proteoforms, including combinations of PTMs or sequence variants on intact proteins. As proteoforms may exert distinct biological effects, future studies using top-down or integrative proteomics strategies will be critical to delineate the specific proteoforms involved in the pathophysiological processes observed. Lastly, adiposomes were isolated from VAT, which may not reflect the full spectrum of EVs circulating in plasma or secreted by other fat depots. Comparative profiling of subcutaneous and circulating adiposomes could provide a more comprehensive view of systemic vesicle signaling in obesity. Despite these limitations, our findings offer a strong foundation for understanding the molecular role of adiposomes in metabolic disease and underscore the value of vesicle-based biomarkers for both mechanistic insight and translational application.

## 5. Conclusions and Future Directions

Our findings open new avenues for understanding adiposomes as dynamic metabolic messengers, vesicles that not only reflect the adipose tissue environment but may actively influence distant organ systems. The observed dysregulation of regulatory proteins such as TTR, ADIPOQ, and PRDX2 suggests these vesicles transmit a systemic “metabolic distress signal,” contributing to tissue-level dysfunction beyond the adipose depot. Integrating adiposome profiling into clinical trials, particularly those evaluating metabolic interventions, could provide novel biomarkers to monitor or predict treatment efficacy. Tracking adiposome proteomic shifts pre- and post-intervention may help determine whether these vesicles transition from carriers of metabolic harm to conveyors of recovery. Lastly, in the era of precision medicine, adiposome proteomics could complement conventional metrics such as BMI and glucose levels to stratify patients better and personalize care. As nutrient-stimulated pharmacotherapy reshapes obesity management, decoding and directing adipose tissue’s vesicular “language” may become a transformative approach to restoring systemic metabolic health.

## Figures and Tables

**Table 1:** Clinical characteristics of the study participants.

	Obese (n = 75)	Control (n = 47)	*p*-Value
Age (years)	37.21 ± 7.69	34.61 ± 8.23	0.0792
BMI (kg/m^2^)	48.53 ± 7.12	25.70 ± 2.99	<0.01
Total Fat percent (%)	52.26 ± 6.80	21.37 ± 3.51	<0.01
Visceral fat mass, kg	2.17 ± 0.69	0.77 ± 0.32	<0.01
Heart rate (b/min)	81.40 ± 12.35	75.85 ± 12.59	0.0198
Systolic blood pressure (mmHg)	132.12 ± 17.71	119.26 ± 11.51	<0.01
Diastolic blood pressure (mmHg)	79.31 ± 9.82	75.30 ± 8.34	0.0185
Fasting insulin (µIU/mL)	15.89 ± 6.15	8.49 ± 1.21	<0.01
Fasting glucose (mg/dL)	105.20 ± 29.81	94.02 ± 11.43	0.0043
HOMA-IR	4.41 ± 2.82	1.96 ± 0.52	<0.01
HbA1C (%)	6.16 ± 1.35	5.38 ± 0.33	<0.01
Total cholesterol (mg/dL)	165.51 ± 31.73	147.89 ± 32.15	0.0041
LDL (mg/dL)	95.64 ± 26.93	82.24 ± 18.03	0.0014
HDL (mg/dL)	43.81 ± 10.28	55.39 ± 17.20	0.0001
Triglycerides (mg/dL)	127.77 ± 64.01	89.65 ± 26.28	<0.01
Nitric oxide (µmol/L)	3.28 ± 1.54	6.41 ± 2.43	<0.01
Alkaline phosphatase (U/L)	80.64 ± 18.04	68.87 ± 21.52	0.0026
AST (U/L)	19.44 ± 12.75	17.78 ± 5.87	0.3340
ALT (U/L)	22.12 ± 19.81	19.83 ± 8.83	0.3854
Total protein (g/dL)	7.22 ± 0.44	7.11 ± 0.52	0.2221
Albumin (g/dL)	4.13 ± 0.36	4.44 ± 0.41	<0.01
Hemoglobin (g/dL)	12.47 ± 1.29	13.93 ± 1.67	<0.01
Brachial FMD (%)	5.78 ± 3.44	17.43 ± 7.34	<0.01
Arteriolar FID (%)	48.32 ± 1.94	76.11 ± 3.21	<0.01
Leptin (ng/mL)	33.93 ± 12.63	10.30 ± 5.93	<0.01
Adiponectin (µg/mL)	5.88 ± 1.87	14.43 ± 7.98	<0.01
Leptin/adiponectin ratio	6.35 ± 3.43	1.06 ± 1.24	<0.01
IL6 (pg/mL)	22.01 ± 12.54	5.34 ± 2.56	<0.01
CRP (mg/L)	3.89 ± 1.51	0.67 ± 0.18	<0.01
Hypertensive	34 (45.3%)	0 (0%)	<0.01
Diabetic	20 (26.7%)	0 (0%)	<0.01
Insulin resistant	36 (48.0%)	0 (0%)	<0.01
Dyslipidemia	34 (45.3%)	0 (0%)	<0.01
Hepatic steatosis	43 (57.3%)	0 (0%)	<0.01
Impaired Vascular Function	46 (61.3%)	0 (0%)	<0.01
Systemic inflammation	49 (65.3%)	12 (26.1%)	<0.01

BMI, Body Mass Index; FMD, Flow-Mediated Dilation; FID, Flow-Induced Dilation; HOMA-IR, Homeostatic Model Assessment of Insulin Resistance; HbA1C, Glycated Hemoglobin; LDL, Low-Density Lipoprotein; HDL, High-Density Lipoprotein; AST, Aspartate Aminotransferase; ALT, Alanine Aminotransferase; CRP, C-Reactive Protein; IL6, Interleukin-6.

**Table 2:** Regulator-target mapping of proteomic abundance.

Target Molecules of Significant Difference	Regulators	Diseases and Functions	Consistency Score	Nodal Total	Known Regulator-Disease/Function Relationship
**ADIPOQ**, CAT, **CRP**, **FGG**, **FN1**, S100A8, SAA1, SERPINF1, **TTR**, VCAM1	TNF	Apoptosis of endothelial cells, Apoptosis of muscle cells, Damage of endothelial tissue, Interaction of mononuclear leukocytes	4.111	15	100% (4/4)
C2, **CFB**, **CRP**, **FN1**, VCAM1	CG (complex), IL1 (family)	Cellular infiltration by leukocytes	3.578	8	100% (2/2)
**ADIPOQ**, **CRP**, **FGG**, **FN1**, **KRT1**, S100A8, SAA1, SERPINF1, VCAM1	TNF	Inflammatory response	−2.667	11	100% (1/1)
**ADIPOQ**, **CRP**, **FGG**, **FN1**, S100A8, SAA1, SERPINF1, VCAM1	TNF	Activation of leukocytes	−3.536	10	100% (1/1)
**ADIPOQ**, **CRP**, **FGG**, **FN1**, S100A8, SAA1, SERPINF1, VCAM1	TNF	Activation of myeloid cells	−3.889	10	100% (1/1)

Adiponectin (ADIPOQ), Catalase (CAT), C-Reactive Protein (CRP), Fibrinogen Gamma Chain (FGG), Fibronectin 1 (FN1), S100 Calcium Binding Protein A8 (S100A8), Serum Amyloid A1 (SAA1), Serpin Family F Member 1 (SERPINF1), Transthyretin (TTR), Vascular Cell Adhesion Molecule 1 (VCAM1), Tumor Necrosis Factor (TNF), Complement Component 2 (C2), Complement Factor B (CFB), Keratin 1 (KRT1), Interleukin 1 family (IL1), Complement Group (CG complex). Bold molecules show a significant difference between obese and lean participants.

## Data Availability

The datasets analyzed during the current study are available from the corresponding author upon reasonable request.

## Supplementary Materials

The following supporting information can be downloaded at: https://www.mdpi.com/article/10.3390/proteomes13030039/s1, Table S1: Regression analysis and association of adiposome lipids with clinical measurements; Table S2: Network-based dissection of obesity-associated molecular dysregulation; Table S3: Excel sheets containing the extracted raw data. Figure S1: Predictive modeling of hypertension and diabetes status using adiposome proteomic profiles; Figure S2: Predictive modeling of steatosis and dyslipidemia status using adiposome proteomic profiles; Figure S3: Original Images for Figure 1D and Figure 1E.
