# Supplementary material for: Adiposome Proteomics Uncover Molecular Signatures of Cardiometabolic Risk in Obese Individuals
Source: Proteomes. 2025 Aug 26;13(3):39. doi: 10.3390/proteomes13030039 (PMC12452415; doi:10.3390/proteomes13030039)
Supplement: Supplementary file 1 [file proteomes-13-00039-s001.zip › proteomes-3743270-Table S1 and S2, Figure S1, S2 and S3.pdf]

Supplementary Table S1. Regression Analysis and Association of Adiposome Lipids with Clinical Measurements

Proteins	Visceral fat (r)	Q value	Proteins	Leptin (r)	Q value	Proteins	brachial FMD (r)	Q value	Proteins	lep/adipo ratio (r)	Q value	Proteins	IL6 (r)	Q value
CFI	**0.416**	**2.04E-06**	**FGB**	**-0.412**	**2.61E-06**	C9	**-0.496**	**7.02E-09**	**TTR**	**-0.366**	**3.75E-05**	**TTR**	**-0.480**	**2.59E-08**
**TTR**	**-0.413**	**2.46E-06**	**FGG**	**-0.394**	**7.78E-06**	**FGG**	**0.432**	**7.42E-07**	TTN	**0.358**	**5.61E-05**	**FGB**	**-0.378**	**1.94E-05**
APOD	**-0.411**	**2.85E-06**	**TTR**	**-0.390**	**1.01E-05**	CFB	**-0.416**	**2.12E-06**	CRP	**0.357**	**5.96E-05**	APOD	**-0.377**	**1.99E-05**
CRP	**0.392**	**8.64E-06**	CRP	**0.382**	**1.53E-05**	**FGB**	**0.416**	**2.14E-06**	**ADIPOQ**	**-0.352**	**7.67E-05**	**FGG**	**-0.370**	**3.00E-05**
**ADIPOQ**	**-0.372**	**2.63E-05**	C9	**0.382**	**1.58E-05**	LRG1	**-0.383**	**1.42E-05**	**FGB**	**-0.348**	**9.28E-05**	APOM	**-0.335**	**1.76E-04**
**IGKV3-15**	**-0.353**	**7.05E-05**	APOC1	**0.363**	**4.31E-05**	F13A1	**0.372**	**2.67E-05**	**FGG**	**-0.334**	**1.84E-04**			
S100A8	**0.349**	**8.77E-05**	SAA4	**0.361**	**4.69E-05**	CRP	**-0.355**	**6.33E-05**						
**FGB**	**-0.335**	**1.75E-04**	CST3	**0.356**	**6.13E-05**	FBLN1	**0.349**	**8.70E-05**						
**FGG**	**-0.334**	**1.79E-04**	**ADIPOQ**	**-0.347**	**9.44E-05**									
APOM	**-0.333**	**1.88E-04**	F13A1	**-0.335**	**1.75E-04**									

Supplementary Table S2. Network-Based Dissection of Obesity-Associated Molecular Dysregulation

Ranking	*Network*	*Molecules in Network*	*Score*	*Top Diseases and Functions*	*Matched molecules with significant difference between obese and lean patients*
1	**Adipokine–Amyloid–Oxidative Stress Interaction**	ADIPOQ,ALP,Amyloid fibril main peptide chains,Amyloid fibrils,APOA4,APOC1,APOD,CAT,COL4,CST3,elastase,FBLN1,Ficolin-rich granule lumen proteins,growth hormone,HDL,HDL/cholesterol,HPX,IL12 (complex),LDL,LRG1,LYZ,N-CoR,NADPH oxidase,NFkB (complex),NR1H,PDGF-BB,PI3K (family),Platelet alpha granule contents,PON1,pro-inflammatory cytokine,SAA1,SERPINF2,SOD,Tertiary granule lumen proteins,TTR	32***	[Hereditary Disorder, Metabolic Disease, Organismal Injury and Abnormalities]	ADIPOQ, APOA4, APOC1^, TTR
2	**Classical Complement Activation Network**	Antigen:IgG:C1Q:2xActivated C1R:SERPING1:2xActivated C1S:SERPING1,Antigen:IgG:C1Q:2xC1R:2xC1S,C-reactive protein pentamer:phosphocholine:C1Q,C1Q (family),C1QB,C1QC,C2,C4,C4 activators,C5-B6,C5/C6/C7,C5/C6/C7/C8/C9,C5b:C6:C7, C8, C9,C6,C7,C8,C9,CD59:C5b-C9,CFB,CFI,CLU:C5b:C6:C7, C8, C9,coagulation factor,Complement,CRP,ERK1/2,F13B,Factor XIII,Fibrin,MAC,MBL2,SERPINF1,TCF,VTN,VTN:C5b:C6:C7,VTN:C5b:C6:C7:C8:C9	29***	[Developmental Disorder, Humoral Immune Response, Inflammatory Response]	C1QB, C9^, CRP^
3	**Toll-like Receptor (TLR)–MyD88 Inflammatory Network**	Activated TLR1:2 or TLR 2:6 heterodimers or TLR4 homodimer,activated TLR2/4:TIRAP:PI(4,5)P2:BTK,AKT,alpha 1 antitrypsin,APOA2,apolipoprotein,APOM,CDK4/6,FAM20C:FAM20C substrates,FGA,FGG,Focal,Integrin alpha IIb beta 3:Fibrin,Integrin alphaDbeta2:fibrin multimer:Mg ,Integrin alphaIIb beta3 ECM ligands,Integrin alphaXbeta2:fibrin multimer,ITI,ITIH1,ITIH2,ITIH4,kallikrein,LDL/cholesterol,PROZ,SAA,SAA4,SERPINA10,SERPINA4,STAT3/STAT3,Talin:RIAM complex:ECM ligands: 2X(Integrin alphaIIb beta3:Inactive (Y530)-SRC),Talin:RIAM complex:ECM ligands: Integrin alphaIIb beta3:Active (Y419)-SRC:PTK2,Talin:RIAM:ECM ligands:2X (Integrin alphaIIb beta3:Active (Y419)-SRC):SYK,TIRAP:PI(4,5)P2:activated TLR2/4,TLR4:LY96:cleaved fibrinogen,VLDL,VLDL/cholesterol	24***	[Cardiac Dysfunction, Cardiovascular Disease, Neurological Disease]	APOA2, FGA, FGG
4	**Cytoskeleton–Adhesion Remodeling Network**	actin,alpha catenin,Ap1,BCR (complex),CG,collagen type i (family),Cornified envelope,Cornified envelope:CDSN,cytokeratin,Early cornified envelope,F9,FCAMR:2xIgA:JCHAIN,FLG fragment:Keratin tonofilament:Desmosome,Hif1,IGHV5-51,IGLV3-10,Immunoglobulin,Integrin alpha 4 beta 1,JCHAIN,keratin,Keratin filament units,Keratin heterodimer,Keratin tonofilament:Desmosome,Keratin tonofilament:Keratin associated proteins polymer,Keratin type II,KRT1,KRT2,KRT6A,KRT9,Late cornified envelope,Mature cornified envelope,PI3K (complex),RAC,trypsin,VCAM1	18**	[Cell Death and Survival, Dermatological Diseases and Conditions, Hereditary Disorder]	KRT1, KRT9
5	**RAF–MAPK Canonical Signaling Network**	activated RAF:scaffold:MAP2K:MAPK,activated RAF:scaffold:MAP2K:MAPK complex:dual mechanism MAP2K inhibitors,activated RAF:scaffold:MAP2K:MAPK:dual mechanism MAPK inhibitors,activated RAF:scaffold:MAP2K:MAPK:single mechanism MAP2K inhibitors,activated RAF:scaffold:MAP2K:MAPK:single mechanism MAPK inhibitors,alpha actinin,BCAR1:Talin:RIAM:ECM ligands:alphaIIb beta3:SRC:PTK2,Collagen type IV,CRK:BCAR1:Talin:RIAM:ECM ligands:alphaIIb beta3:SRC:PTK2,F13A1,FGB,Fibrinogen,FN1,FRMPD1,GRB2 bound to pPTK2 in Focal adhesion,GRB2:SOS:PTK2:Focal adhesion,IRAK1/or IRAK2:IRAK4:MyD88 oligomer:TIRAP:activated TLR,IRAK1:IRAK4:oligo-MyD88:TIRAP:activated TLR,IRAK2:IRAK4:oligo-MyD88:Mal:activated TLR,IRAK4:oligo-MyD88:TIRAP:activated TLR,ITGA2B/ITGB3,JNK,MyD88:TIRAP:BTK:activated TLR2/4,NFAT (complex),PDGF (complex),PKG,Platelet releasate cytosolic proteins,PRDX2,RAF/MAPK scaffolds,TAGLN2,TLN1,TRAF6:IRAK1:IRAK4:oligo-MyD88:TIRAP:activated TLR,TRAF6:IRAK2 :IRAK4:oligo-MyD88:TIRAP:activated TLR,transglutaminase,TTN	16**	[Cancer, Hematological Disease, Hematological System Development and Function]	FGB, FN1, PRDX2, TLN1
6	**B Cell Receptor Signaling and Immunoglobulin Activation Network**	2xIgA:JCHAIN,Antigen:BCR:SYK,Antigen:BCR:SYK:BLNK:CIN85:GRB2:SOS1,Antigen:BCR:SYK:BLNK:CIN85:GRB2:SOS1:BTK:NCK1:VAV1:PLCG2,BCR Reactome,BTK:BTK inhibitors,C1Q,CD22:Antigen:BCR,chemokine,Collagen type I (complex),Collagen(s),Complement component 1,CREB,ERK,fibrinogen (family),focal adhesion kinase,IC,IgA,IgA:Alpha-1-Microglobulin,IgG,IGHM,IGKV3-15,IGKV3-20,IgM,IL1,IL12 (family),Integrin,Laminin (complex),LYN, SYK,NFkB (family),PKA,PRG4,proinsulin,PTPN6:CD22:Antigen:BCR,TGF beta	7**	[Hereditary Disorder, Organismal Injury and Abnormalities, Skeletal and Muscular Disorders]	IGHM, IGKV3-15
7	**T Cell and MAPK-linked Immune-Stress Response Network**	11-dehydrocorticosterone,Adducin,AMPK,CD3 (complex),CK2,CLEC9A,CNTN6,Cyp2j9,cytokine,D-mannose,EEF1A,ENO1,enolase,F Actin,FSH,Gzmb (includes others),H/K/NRAS,histone H3,histone H4,IFN beta,IgE,IKB,insulin,MAPK,MEK,MHC II (complex),N,N-dimethylarginine,p38 MAPK,PKC,RNA polymerase II,S100A12,S100A8,SRC (family),TCR (complex),VEGF	3**	[Cardiovascular Disease, Cell-To-Cell Signaling and Interaction, Inflammatory Response]	-
8	**Redox–Autophagy–ER Stress Regulatory Network**	ARL10,ATG16L1,CCDC168,CUL1,CYBA,CYBB,H1-5,HSPA5,MST1R,NUP43,RPS24,SLC30A1,TMT1A,USO1,USP34	2*	[Hereditary Disorder, Immunological Disease, Inflammatory Disease]	-
9	**Contactin–NOTCH–Neuronal Interaction Network**	ADGRA2,ADGRA3,ARMC1,ARRDC5,C12orf75,CD276,CEACAM21,CNTN1,CNTN1:NOTCH1:DTX,CNTN1:NOTCH2,CNTN2,CNTNAP1,Contactin1:CASPR,DGUOK,GJB7,KYAT3,L1:CNTN1,MAGEA3,MEGF9,MPP1,Neurofascin:CNTN1:CASPR,Notch Signaling,PALM3,PCDH9,PGAP6,PHLPP2,SCN1B,SCN3A,SIGLECL1,Signaling by NOTCH2,sodium channel,SORCS2,THYN1,TMEM132D,voltage-gated sodium channel	1	[Cell-To-Cell Signaling and Interaction, Nervous System Development and Function, Neurological Disease]	-

* Significant (p value =0.01)

** Strongly significant (p value <0.001)

*** Extremely significant (p value $<10^{-20}$)

^ Upregulated molecules, else are downregulated

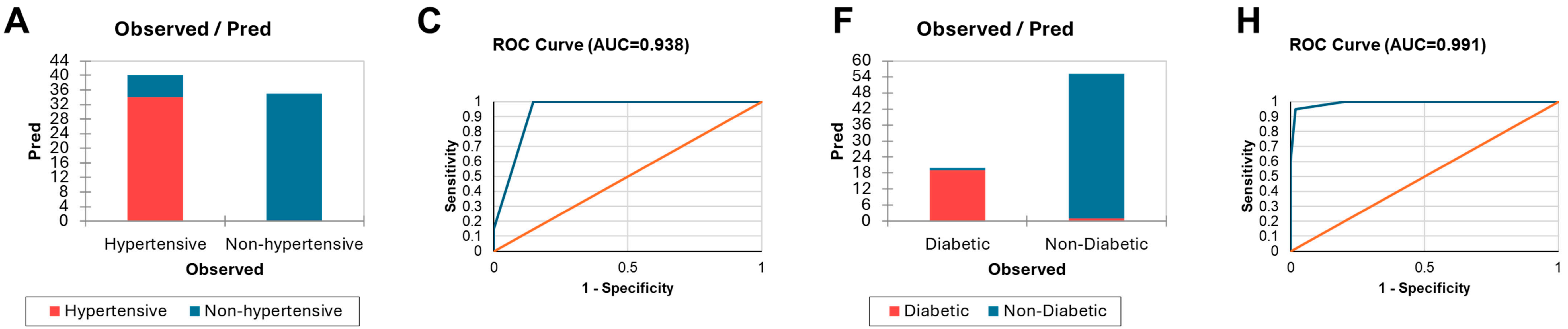

B

from \ to	Hypertensive	Non-hypertensive	Total	% correct
Hypertensive	34	0	34	100.00%
Non-hypertensive	6	35	41	85.37%
Total	40	35	75	92.00%

G

from \ to	Diabetic	Non-Diabetic	Total	% correct
Diabetic	19	1	20	95.00%
Non-Diabetic	1	54	55	98.18%
Total	20	55	75	97.33%

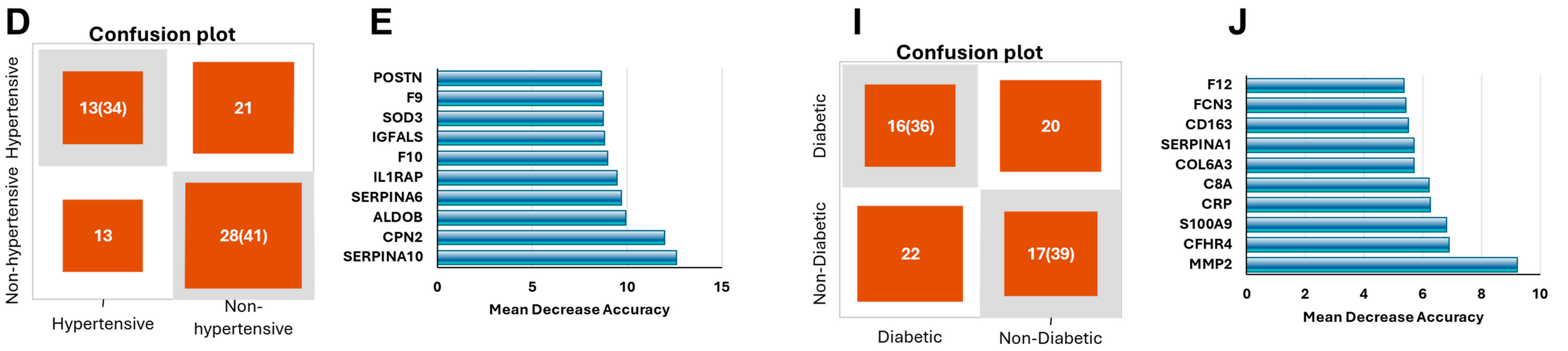

Supplementary Figure S1. Predictive modelling of hypertension and diabetes status using adiposome proteomic profiles. (A-B) A decision tree classification model, bar plot, and table showing model accuracy in the hypertensive and non-hypertensive groups. (C) Receiver Operating Characteristic (ROC) curve for the decision tree model. (D) Confusion matrix illustrating correct and incorrect classifications. (E) Variable importance plot from the random forest model. (F-G) A decision tree classification model, bar plot, and table showing model accuracy in the diabetic and non-diabetic groups. (H) Receiver Operating Characteristic (ROC) curve for the decision tree model. (I) Confusion matrix illustrating correct and incorrect classifications. (J) Variable importance plot from the random forest model.

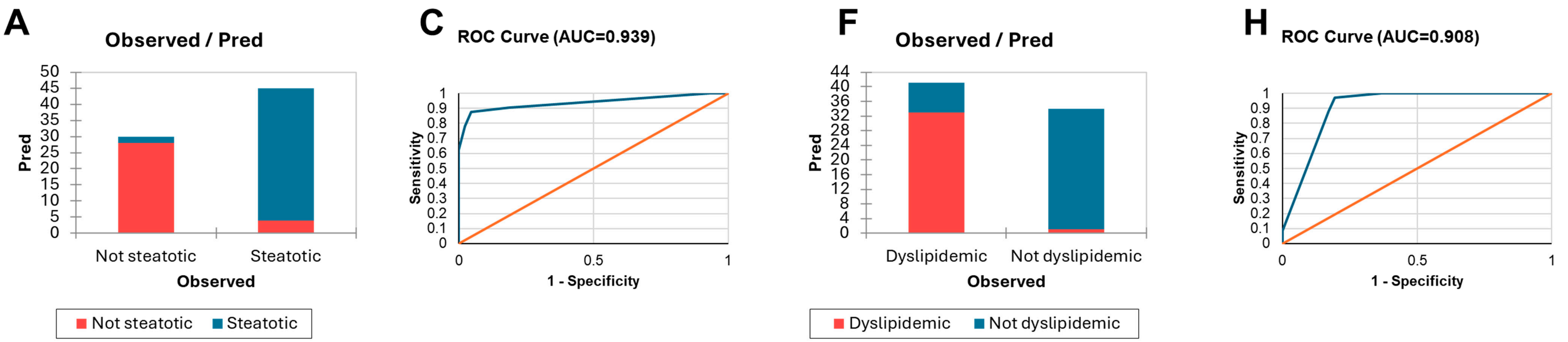

B

from \ to	Not steatotic	Steatotic	Total	% correct
Not steatotic	28	4	32	87.50%
Steatotic	2	41	43	95.35%
Total	30	45	75	92.00%

G

from \ to	Dyslipidemic	Not dyslipidemic	Total	% correct
Dyslipidemic	33	1	34	97.06%
Not dyslipidemic	8	33	41	80.49%
Total	41	34	75	88.00%

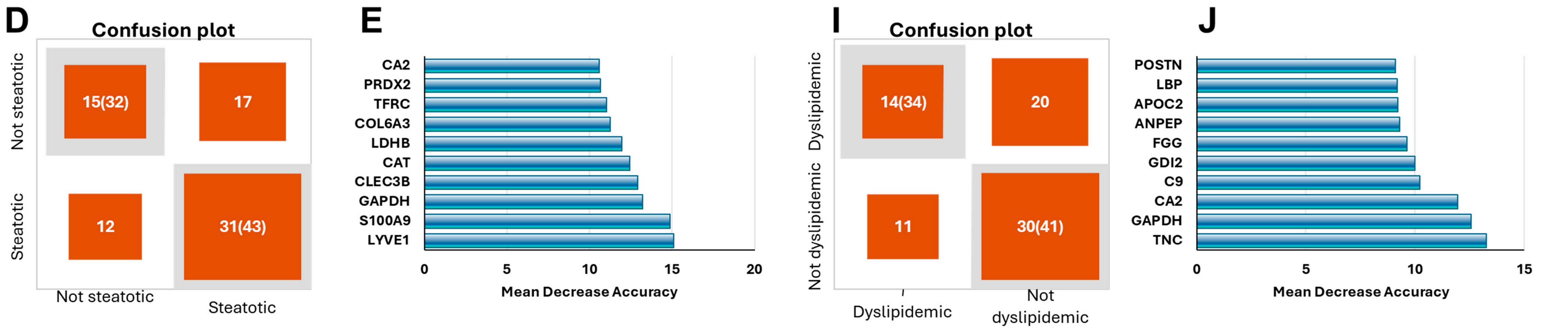

Supplementary Figure S2. Predictive modelling of liver steatotic and dyslipidemic status using adiposome proteomic profiles. (A-B) A decision tree classification model, bar plot, and table showing model accuracy in the steatotic vs. non-steatotic groups. (C) Receiver Operating Characteristic (ROC) curve for the decision tree model. (D) Confusion matrix illustrating correct and incorrect classifications. (E) Variable importance plot from the random forest model. (F-G) A decision tree classification model, bar plot, and table showing model accuracy in the dyslipidemic and non-dyslipidemic groups. (H) Receiver Operating Characteristic (ROC) curve for the decision tree model. (I) Confusion matrix illustrating correct and incorrect classifications. (J) Variable importance plot from the random forest model.

Figure 1D

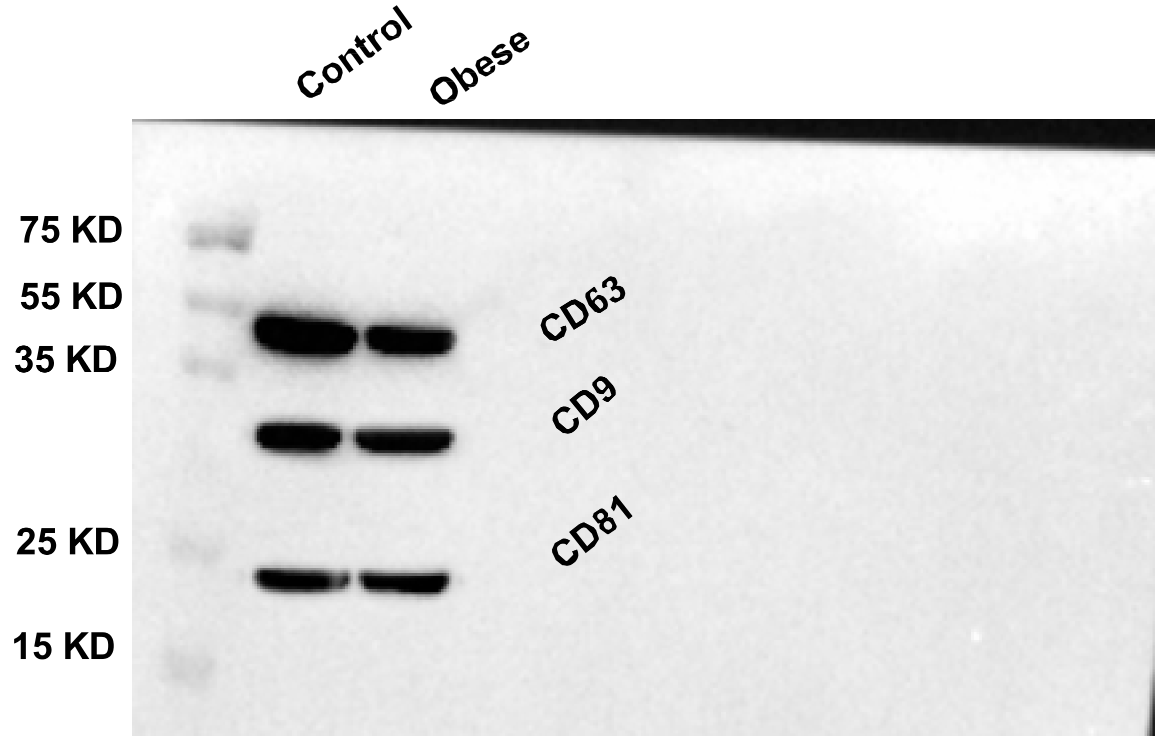

Figure 1E

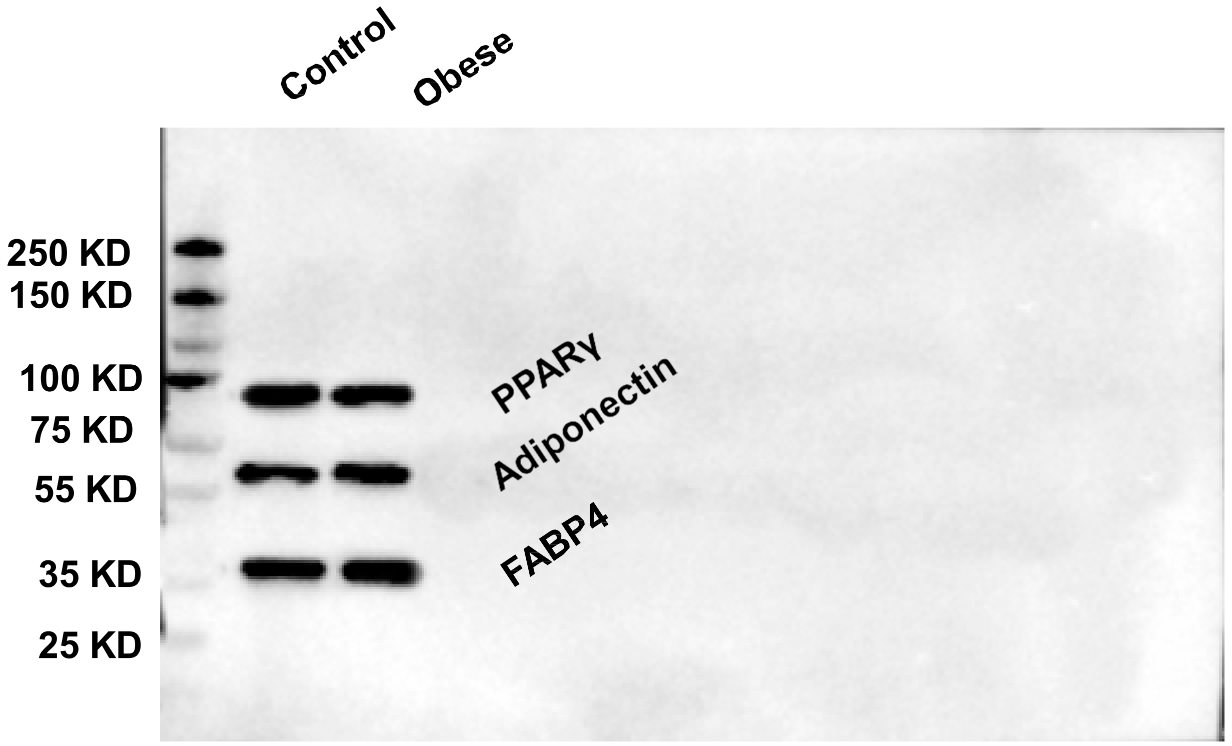

Supplementary Figure S3. Original Images for Figure 1D and Figure 1E
